# Supplementary figures and images for: DSTYK phosphorylates STING at late endosomes to promote STING signaling
Source: EMBO Rep. 2025 Feb 20;26(6):1620–46. doi: 10.1038/s44319-025-00394-9 (PMC11933320; doi:10.1038/s44319-025-00394-9)

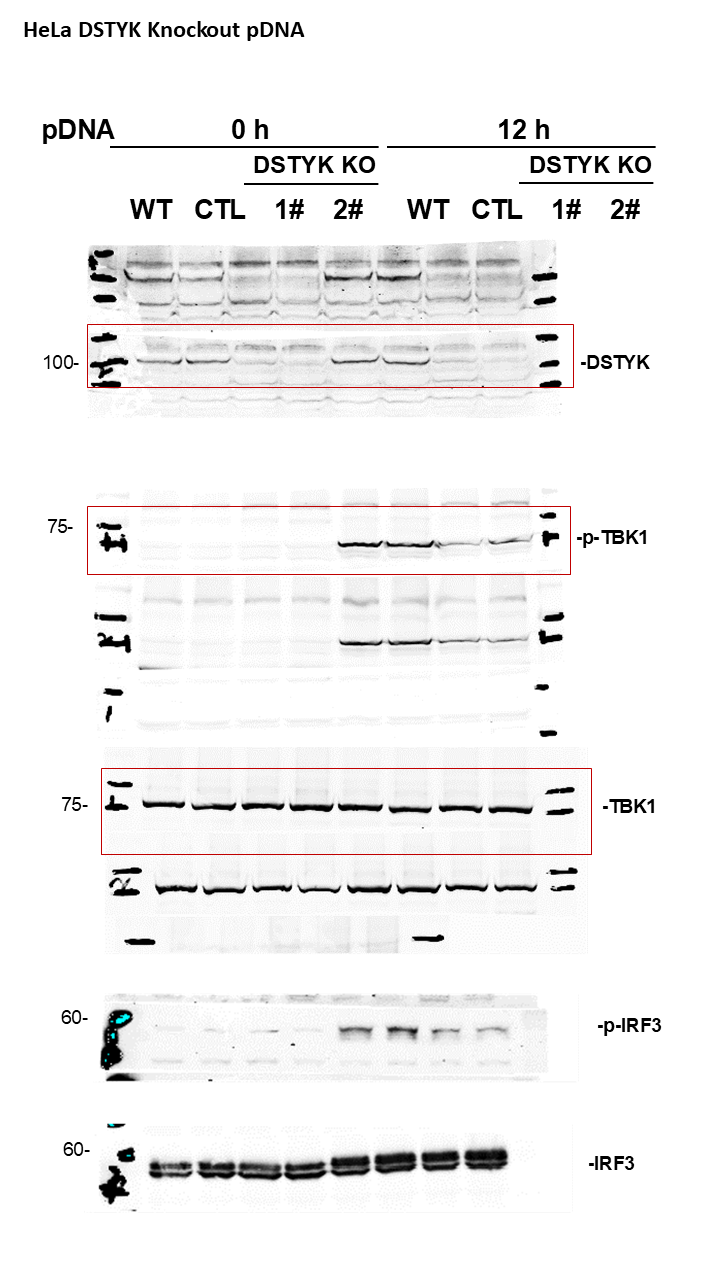

Supplement: Supplementary file 2 — Source data Fig. 1 [file 44319_2025_394_MOESM2_ESM.zip › Figure 1/Figure 1C/Figure 1C-1.TIF]

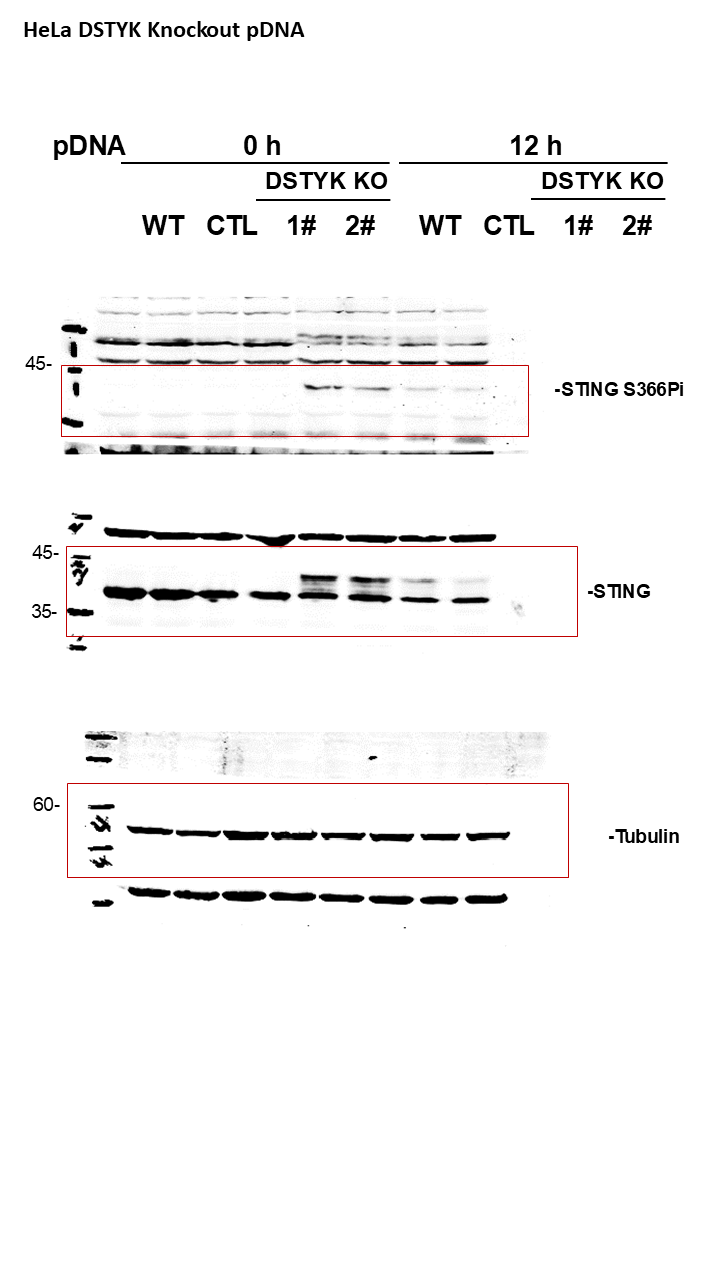

Supplement: Supplementary file 2 — Source data Fig. 1 [file 44319_2025_394_MOESM2_ESM.zip › Figure 1/Figure 1C/Figure 1C-2.TIF]

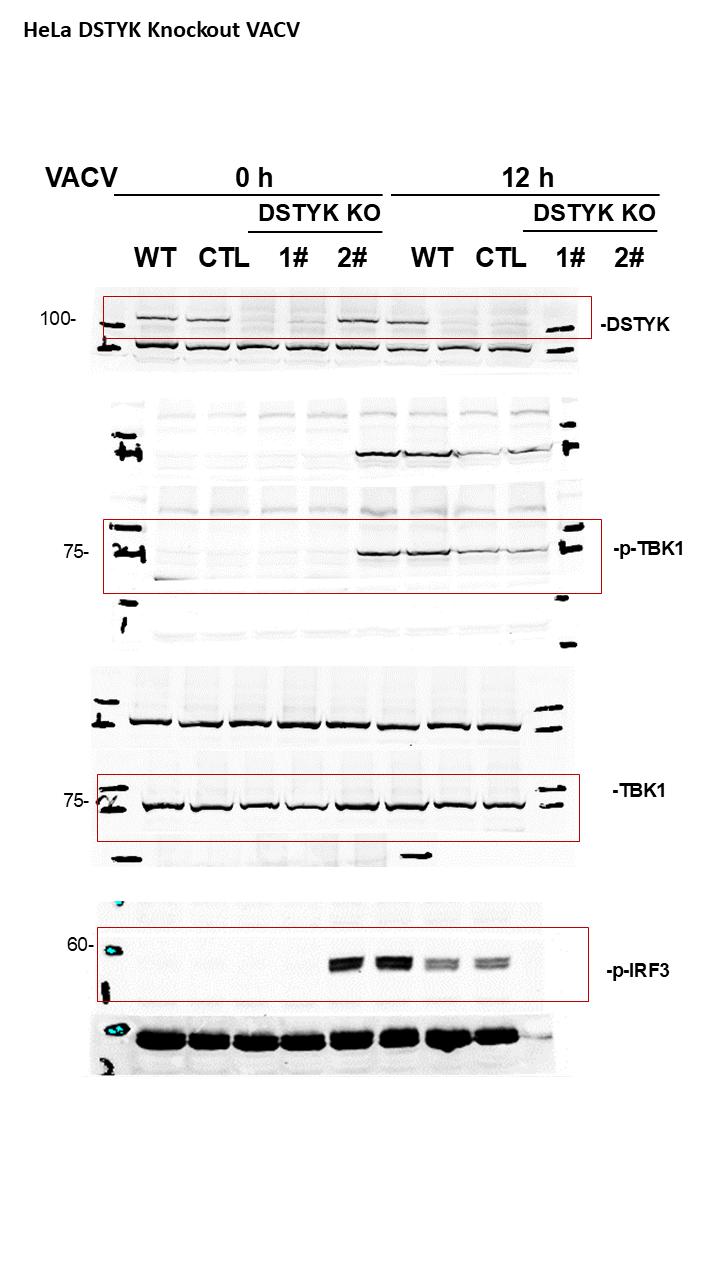

Supplement: Supplementary file 2 — Source data Fig. 1 [file 44319_2025_394_MOESM2_ESM.zip › Figure 1/Figure 1D/Figure 1D-1.TIF]

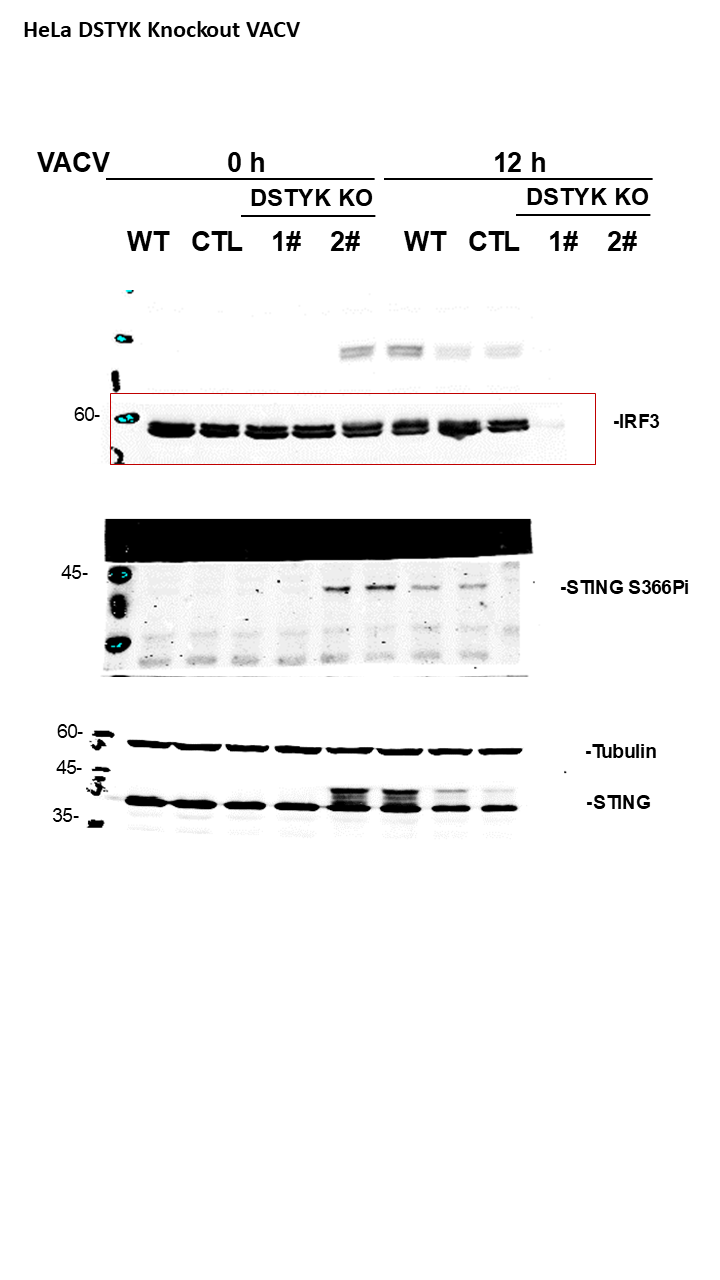

Supplement: Supplementary file 2 — Source data Fig. 1 [file 44319_2025_394_MOESM2_ESM.zip › Figure 1/Figure 1D/Figure 1D-2.TIF]

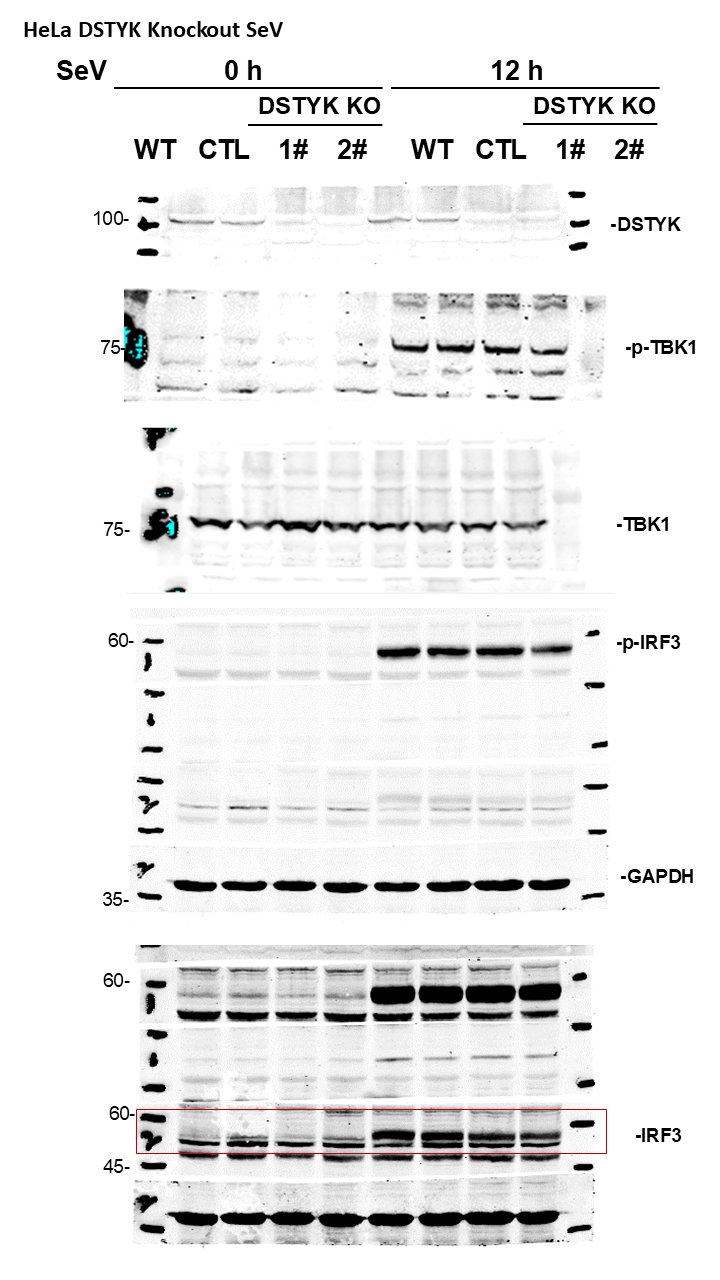

Supplement: Supplementary file 2 — Source data Fig. 1 [file 44319_2025_394_MOESM2_ESM.zip › Figure 1/Figure 1E/Figure 1E.TIF]

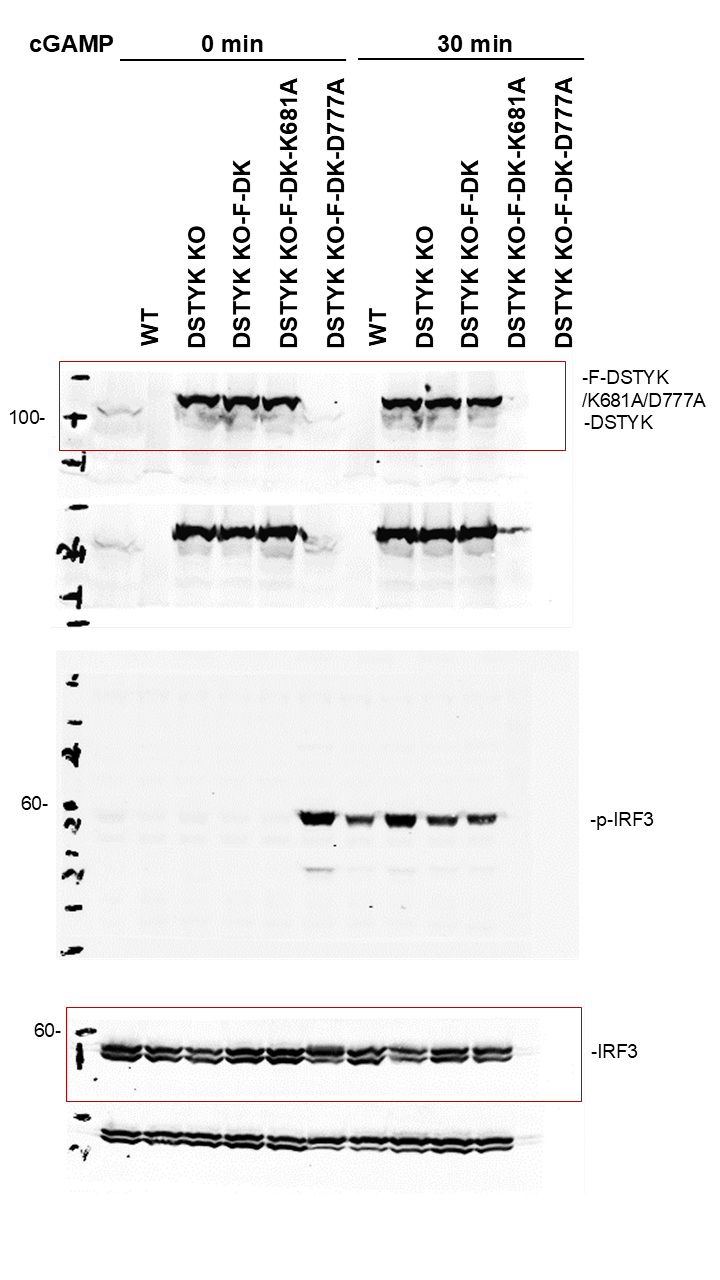

Supplement: Supplementary file 3 — Source data Fig. 2 [file 44319_2025_394_MOESM3_ESM.zip › Figure 3/Figure 3B/Figure 3B-1.TIF]

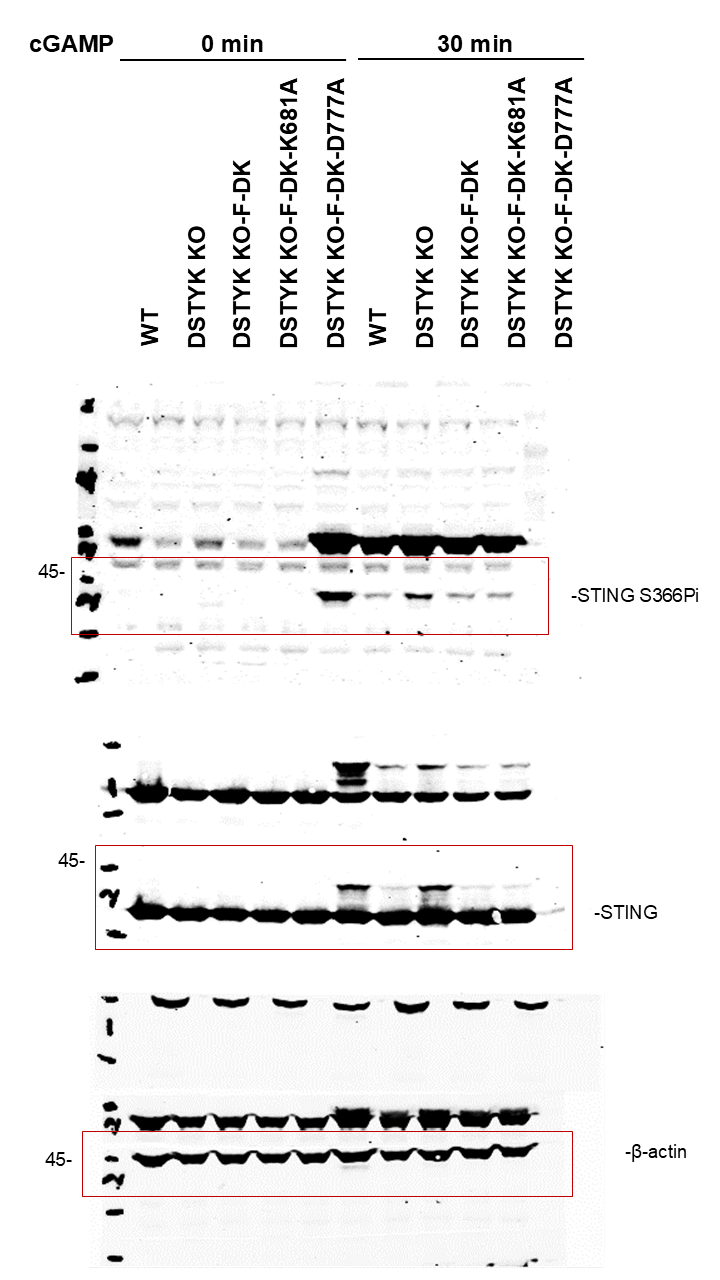

Supplement: Supplementary file 3 — Source data Fig. 2 [file 44319_2025_394_MOESM3_ESM.zip › Figure 3/Figure 3B/Figure 3B-2.TIF]

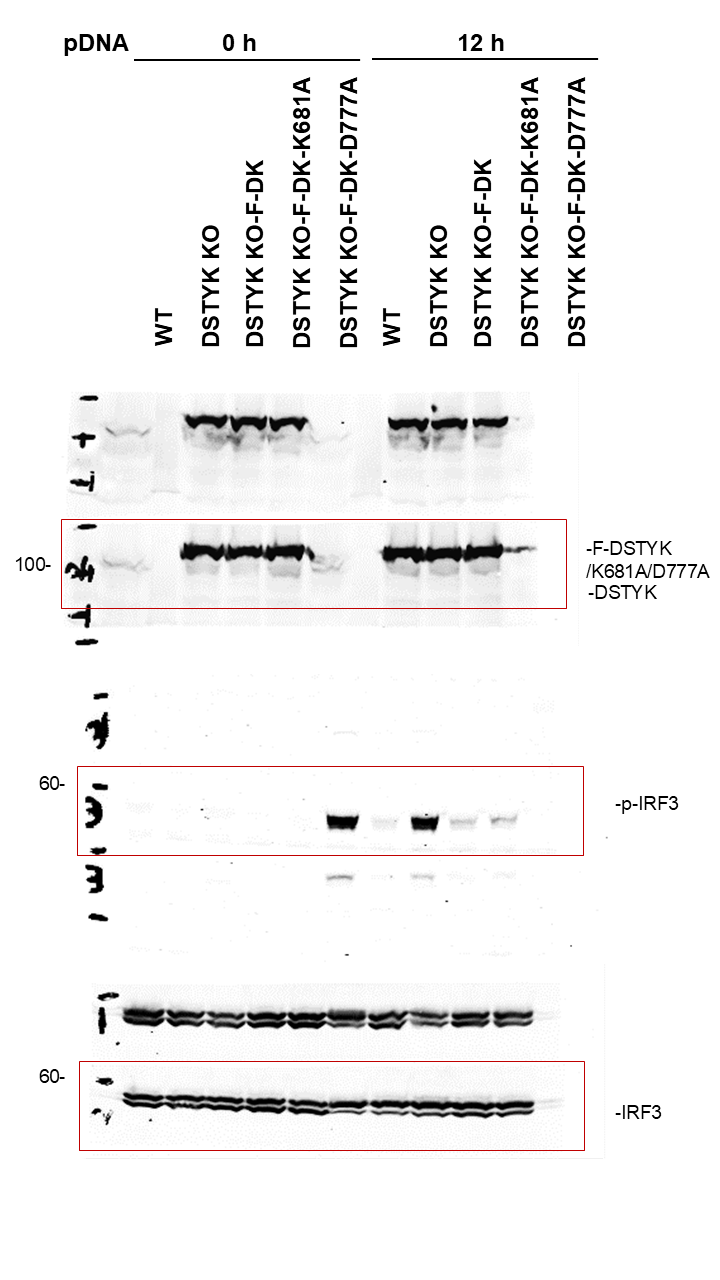

Supplement: Supplementary file 3 — Source data Fig. 2 [file 44319_2025_394_MOESM3_ESM.zip › Figure 3/Figure 3C/Figure 3C-1.TIF]

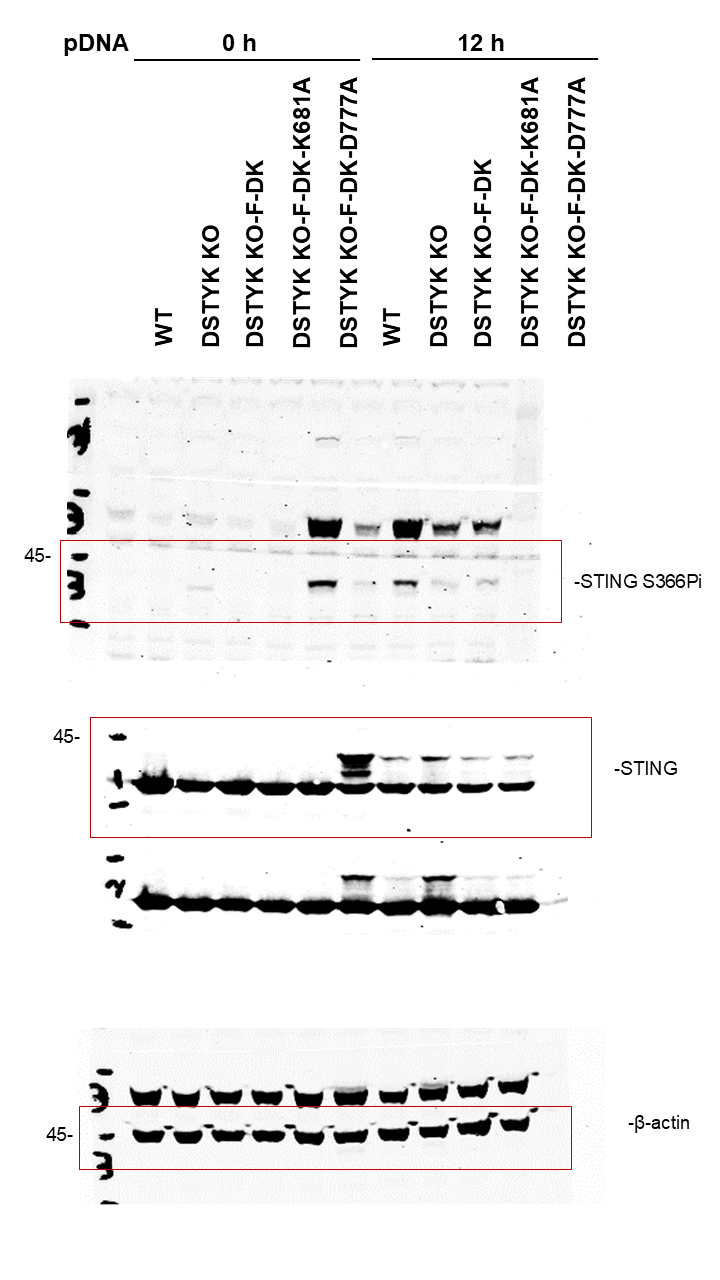

Supplement: Supplementary file 3 — Source data Fig. 2 [file 44319_2025_394_MOESM3_ESM.zip › Figure 3/Figure 3C/Figure 3C-2.TIF]

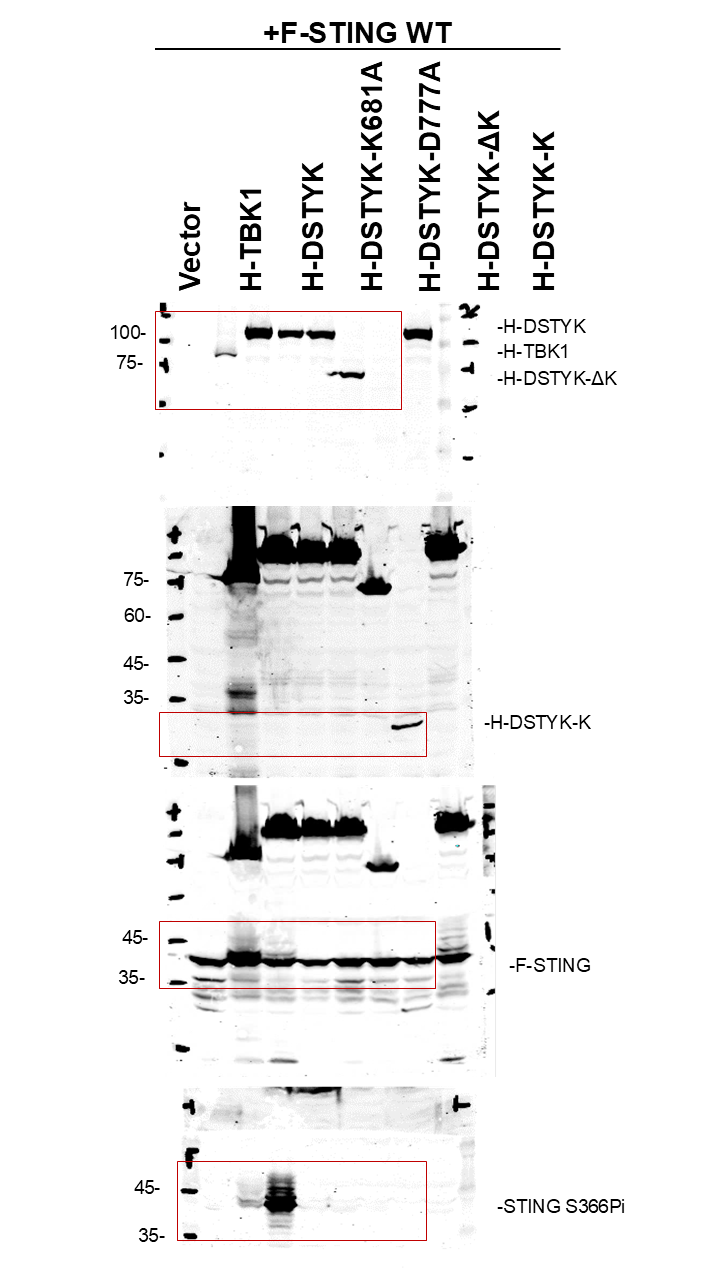

Supplement: Supplementary file 3 — Source data Fig. 2 [file 44319_2025_394_MOESM3_ESM.zip › Figure 3/Figure 3D/Figure 3D.TIF]

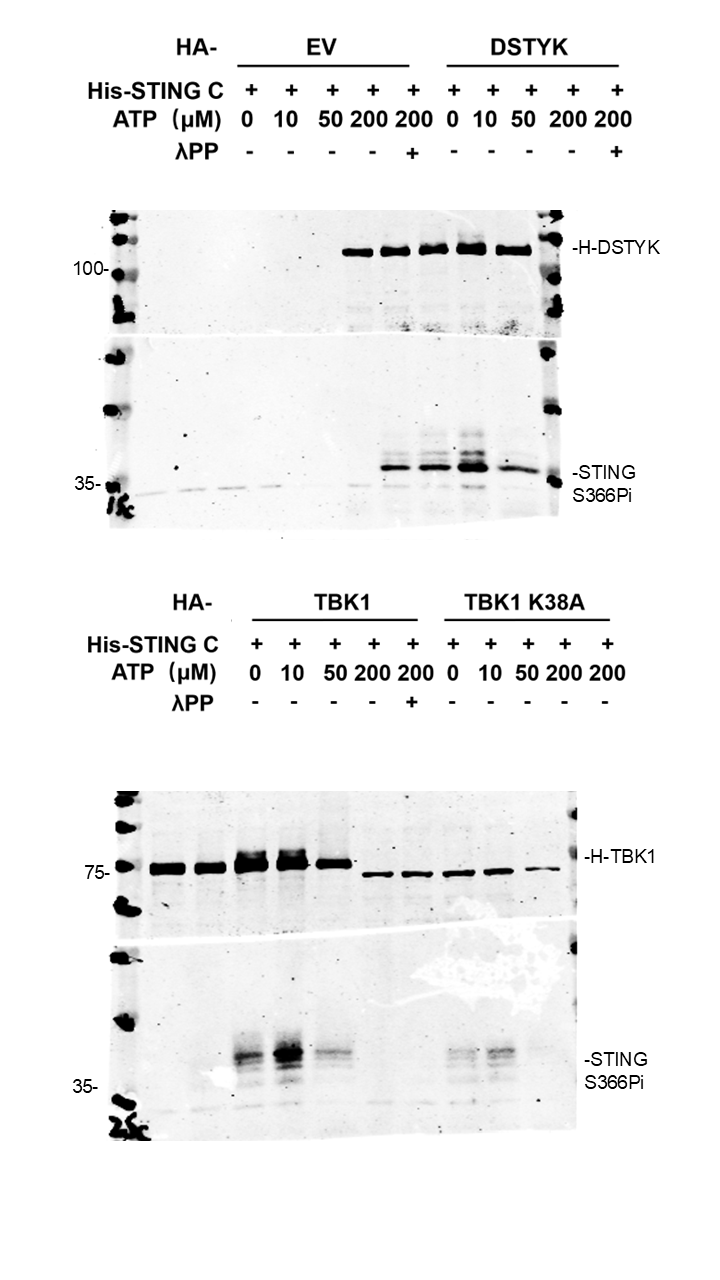

Supplement: Supplementary file 3 — Source data Fig. 2 [file 44319_2025_394_MOESM3_ESM.zip › Figure 3/Figure 3E/Figure 3E-1.TIF]

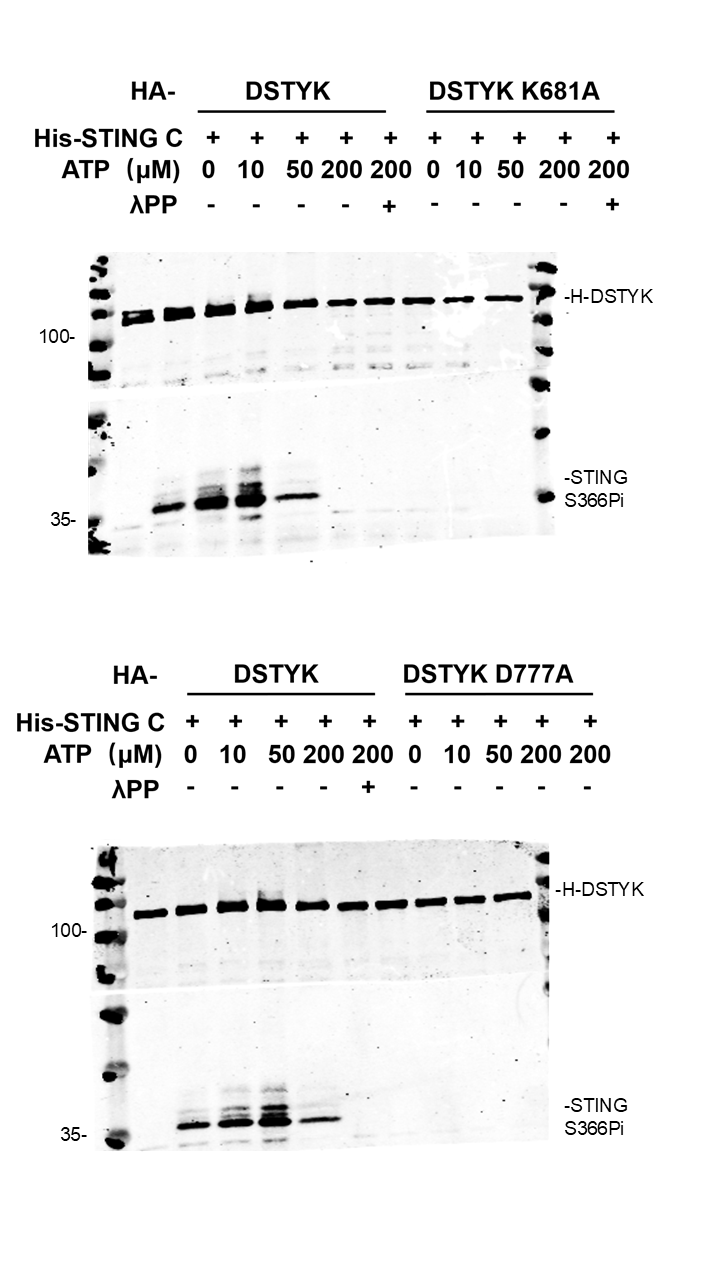

Supplement: Supplementary file 3 — Source data Fig. 2 [file 44319_2025_394_MOESM3_ESM.zip › Figure 3/Figure 3E/Figure 3E-2.TIF]

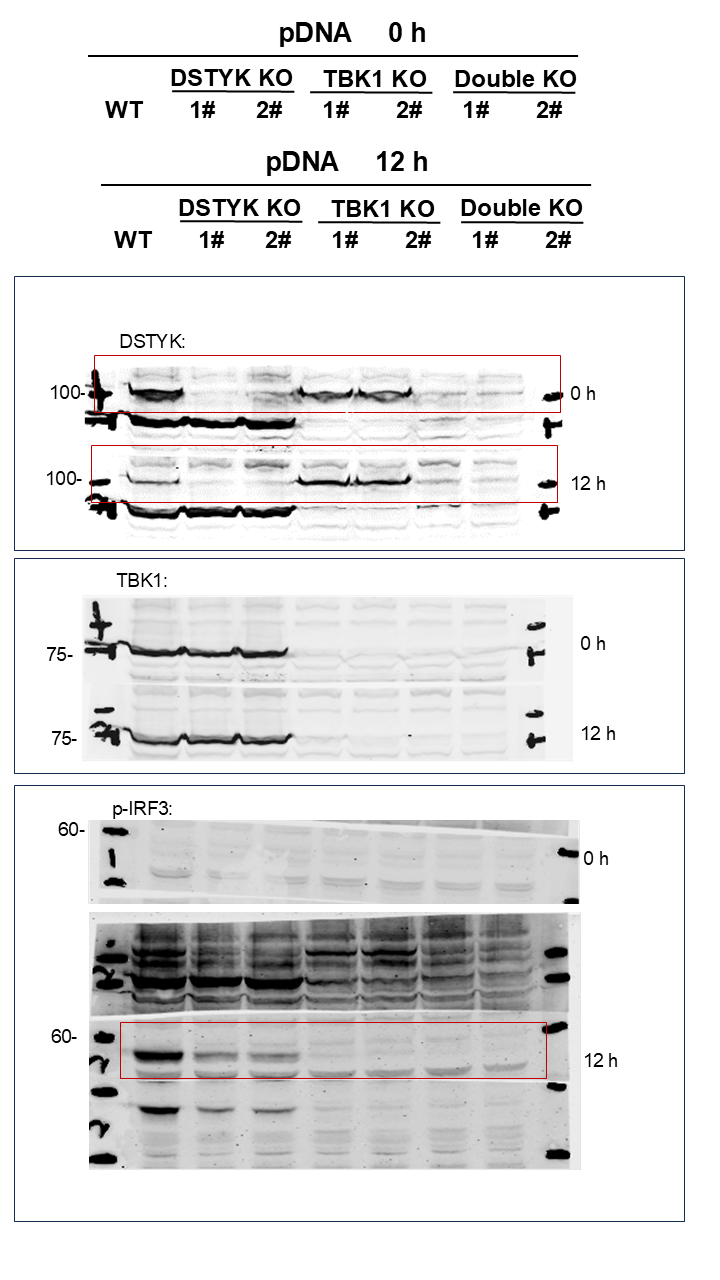

Supplement: Supplementary file 4 — Source data Fig. 3 [file 44319_2025_394_MOESM4_ESM.zip › Figure 4/Figure 4A/Figure 4A-1.tif]

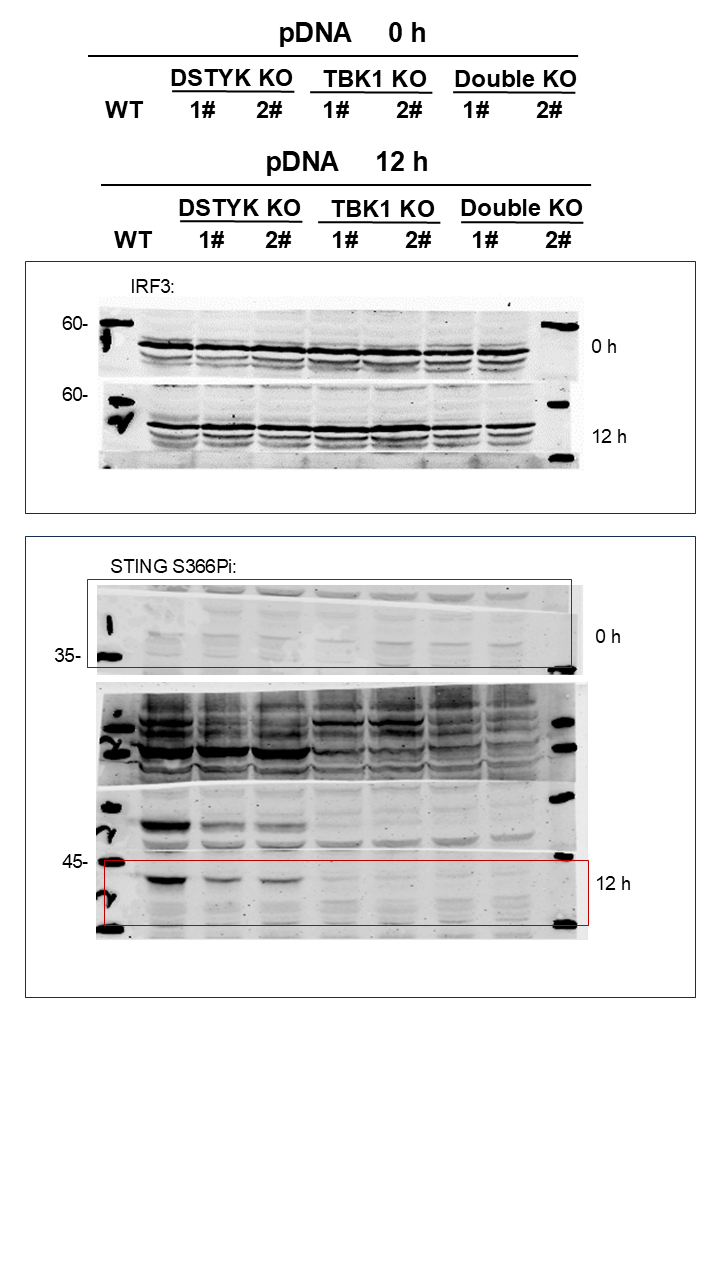

Supplement: Supplementary file 4 — Source data Fig. 3 [file 44319_2025_394_MOESM4_ESM.zip › Figure 4/Figure 4A/Figure 4A-2.tif]

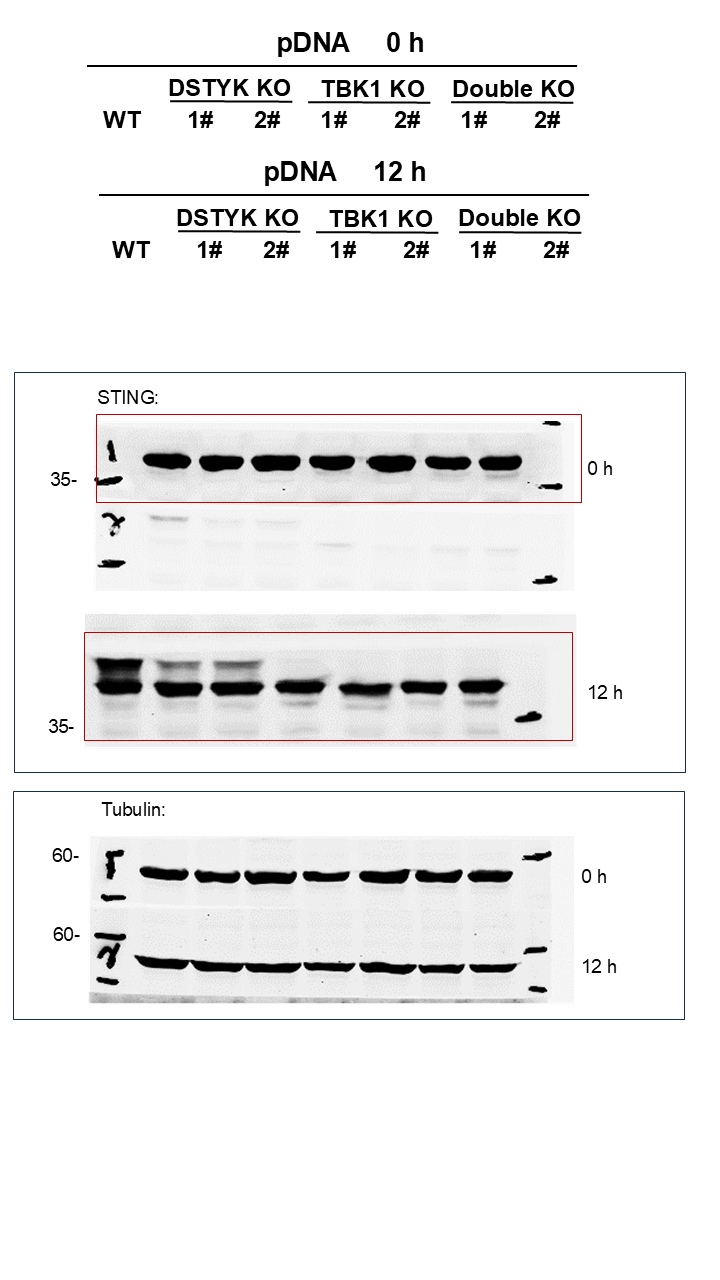

Supplement: Supplementary file 4 — Source data Fig. 3 [file 44319_2025_394_MOESM4_ESM.zip › Figure 4/Figure 4A/Figure 4A-3.tif]

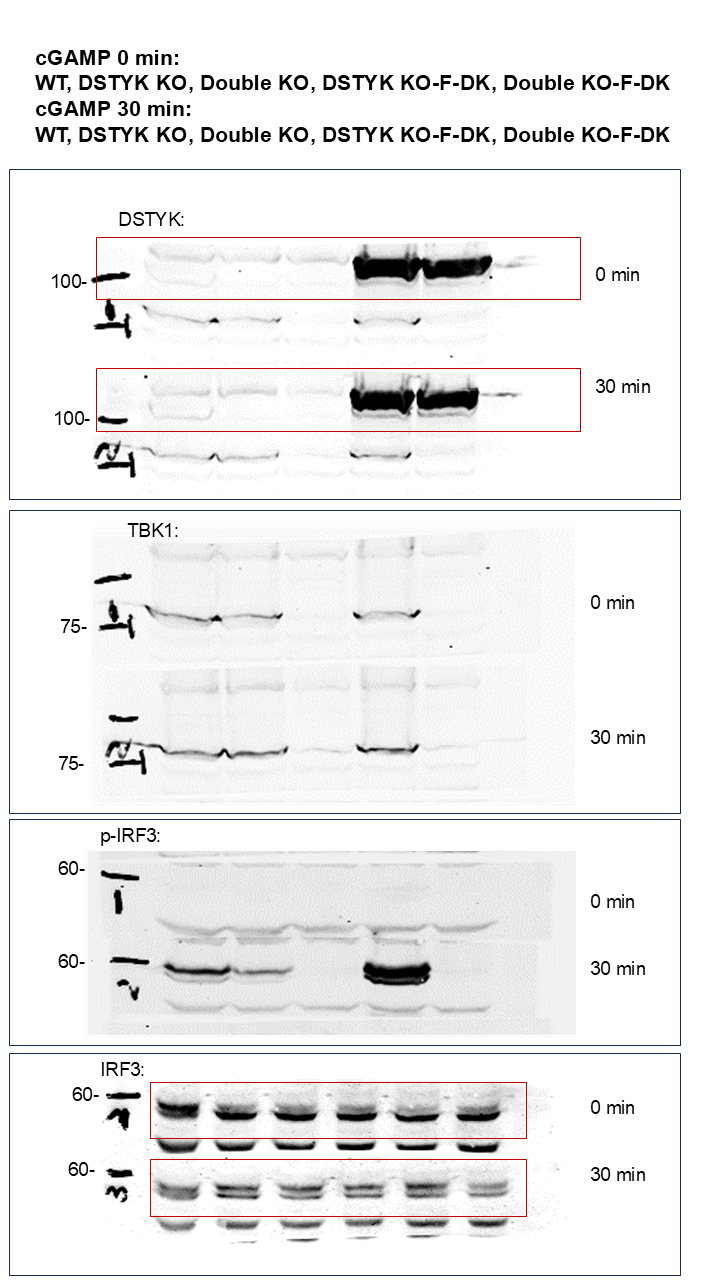

Supplement: Supplementary file 4 — Source data Fig. 3 [file 44319_2025_394_MOESM4_ESM.zip › Figure 4/Figure 4C/Figure 4C-1.TIF]

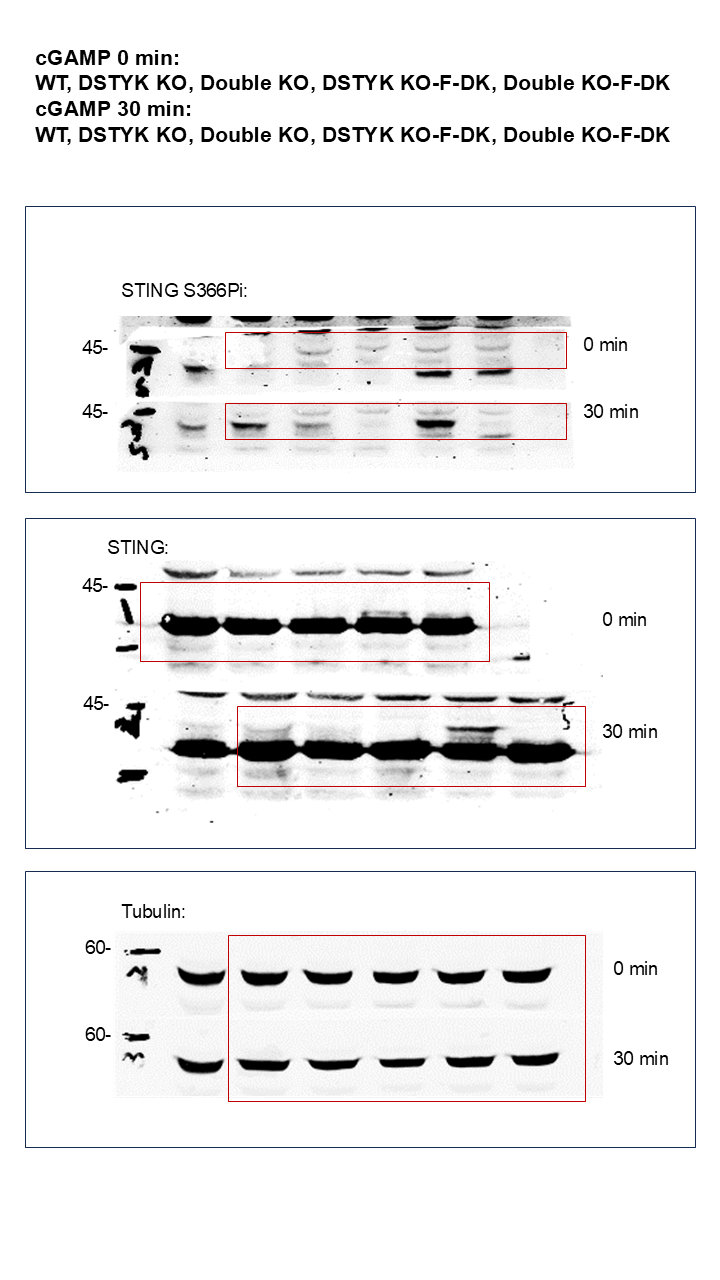

Supplement: Supplementary file 4 — Source data Fig. 3 [file 44319_2025_394_MOESM4_ESM.zip › Figure 4/Figure 4C/Figure 4C-2.TIF]

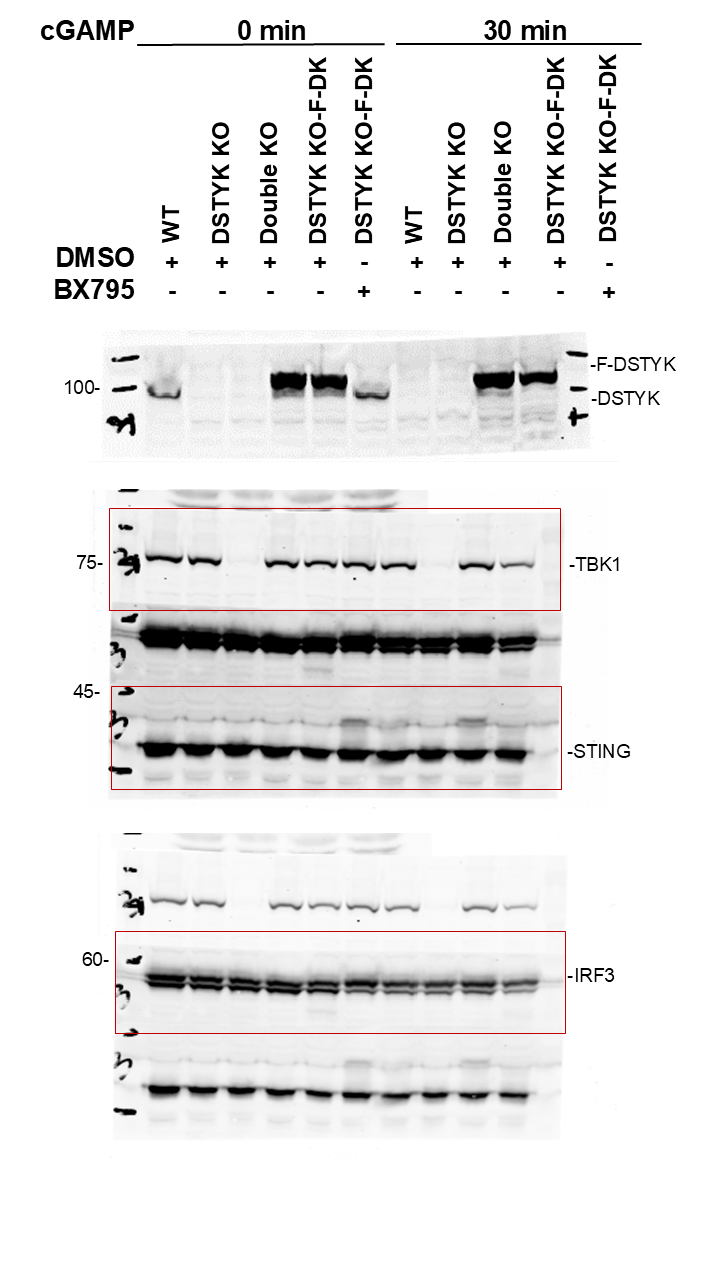

Supplement: Supplementary file 4 — Source data Fig. 3 [file 44319_2025_394_MOESM4_ESM.zip › Figure 4/Figure 4E/Figure 4E-1.TIF]

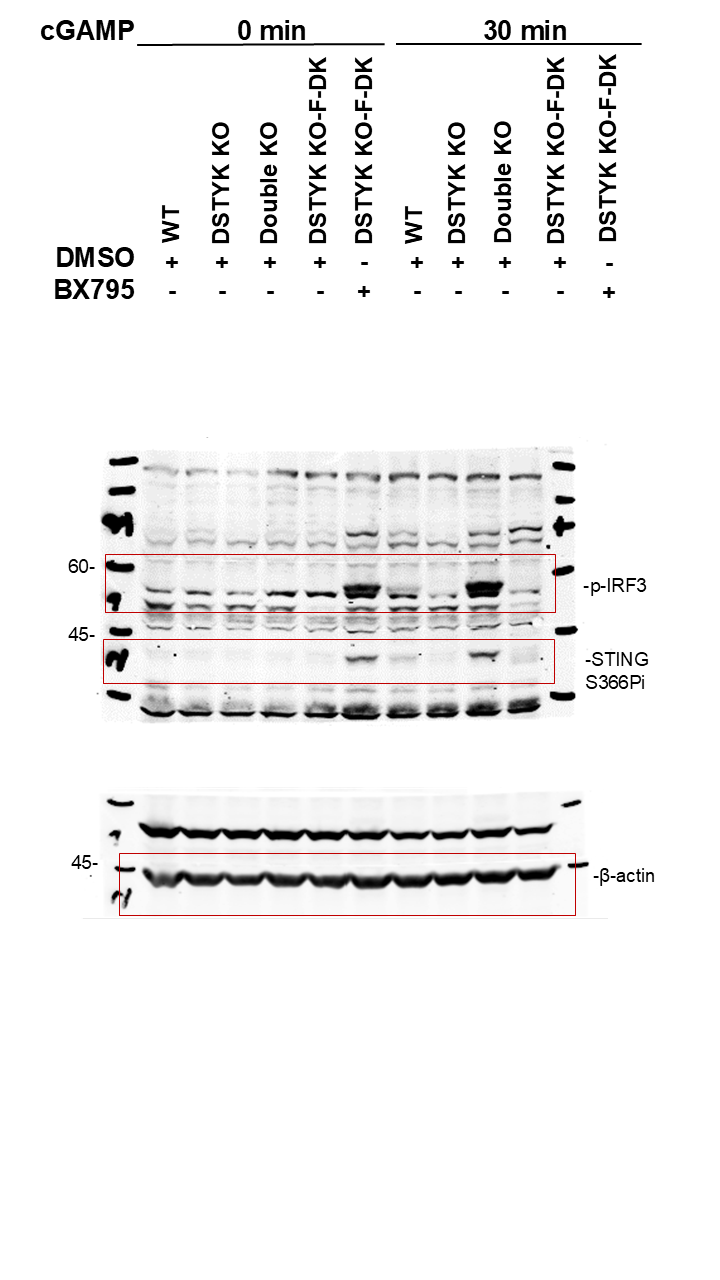

Supplement: Supplementary file 4 — Source data Fig. 3 [file 44319_2025_394_MOESM4_ESM.zip › Figure 4/Figure 4E/Figure 4E-2.TIF]

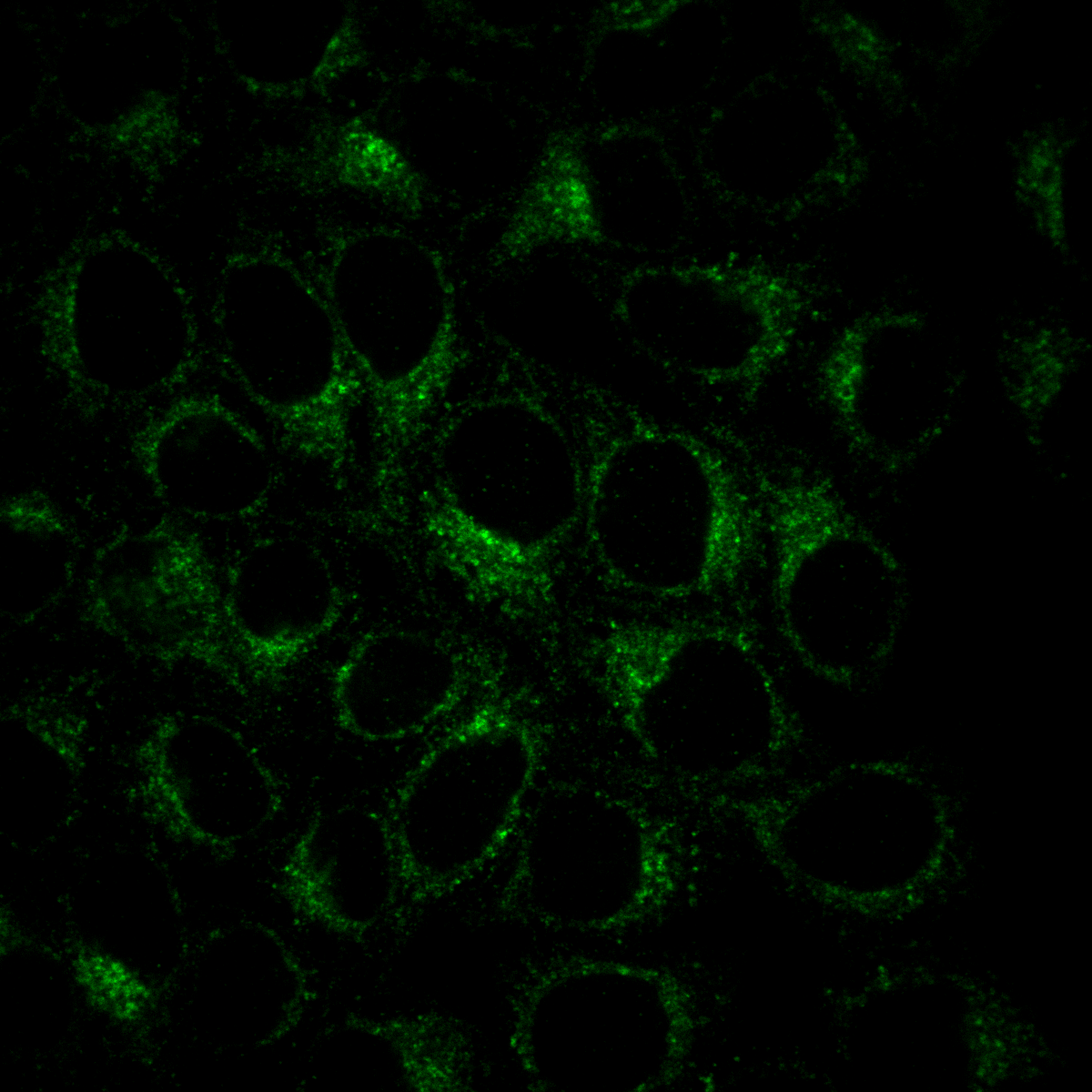

Supplement: Supplementary file 4 — Source data Fig. 3 [file 44319_2025_394_MOESM4_ESM.zip › Figure 4/Figure 4H/DMSO STING+Rab7 0 h/HeLa DMSO P0 STING RAB7-3_w2CSU GFP.TIF]

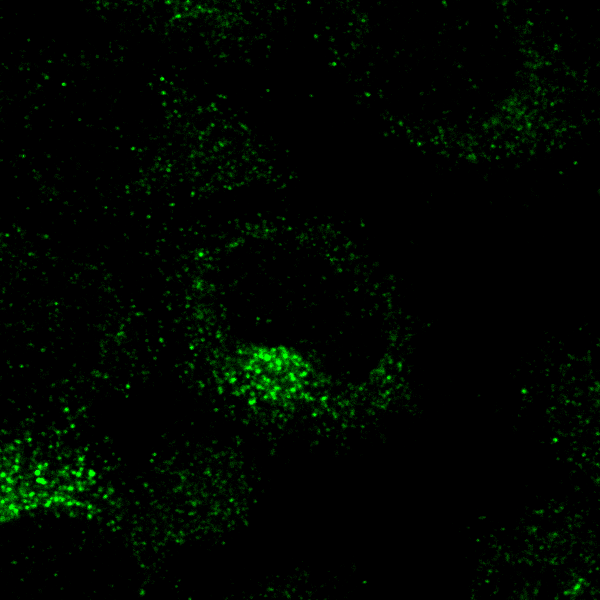

Supplement: Supplementary file 5 — Source data Fig. 4 [file 44319_2025_394_MOESM5_ESM.zip › Figure 5/Figure 5B/F-DK STING TBK1 24-5_w1CSU GFP.TIF]

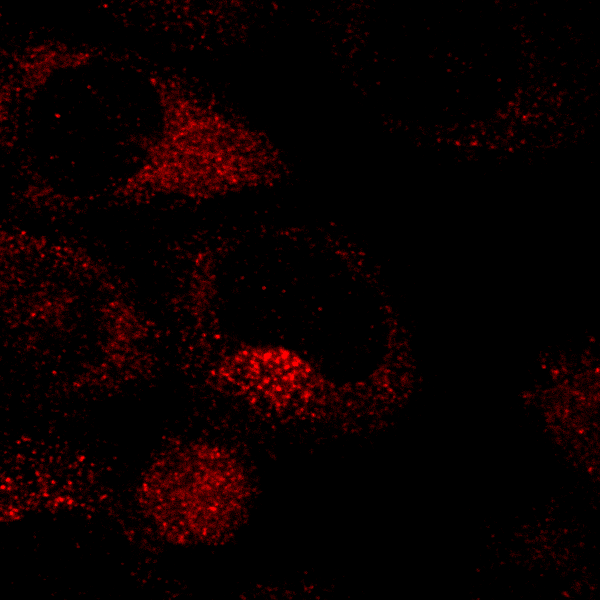

Supplement: Supplementary file 5 — Source data Fig. 4 [file 44319_2025_394_MOESM5_ESM.zip › Figure 5/Figure 5B/F-DK STING TBK1 24-5_w2CSU RFP.TIF]

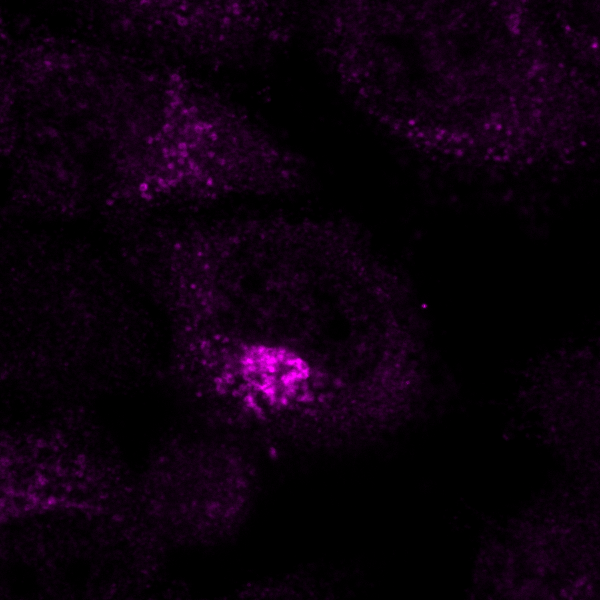

Supplement: Supplementary file 5 — Source data Fig. 4 [file 44319_2025_394_MOESM5_ESM.zip › Figure 5/Figure 5B/F-DK STING TBK1 24-5_w3CSU Cy5.TIF]

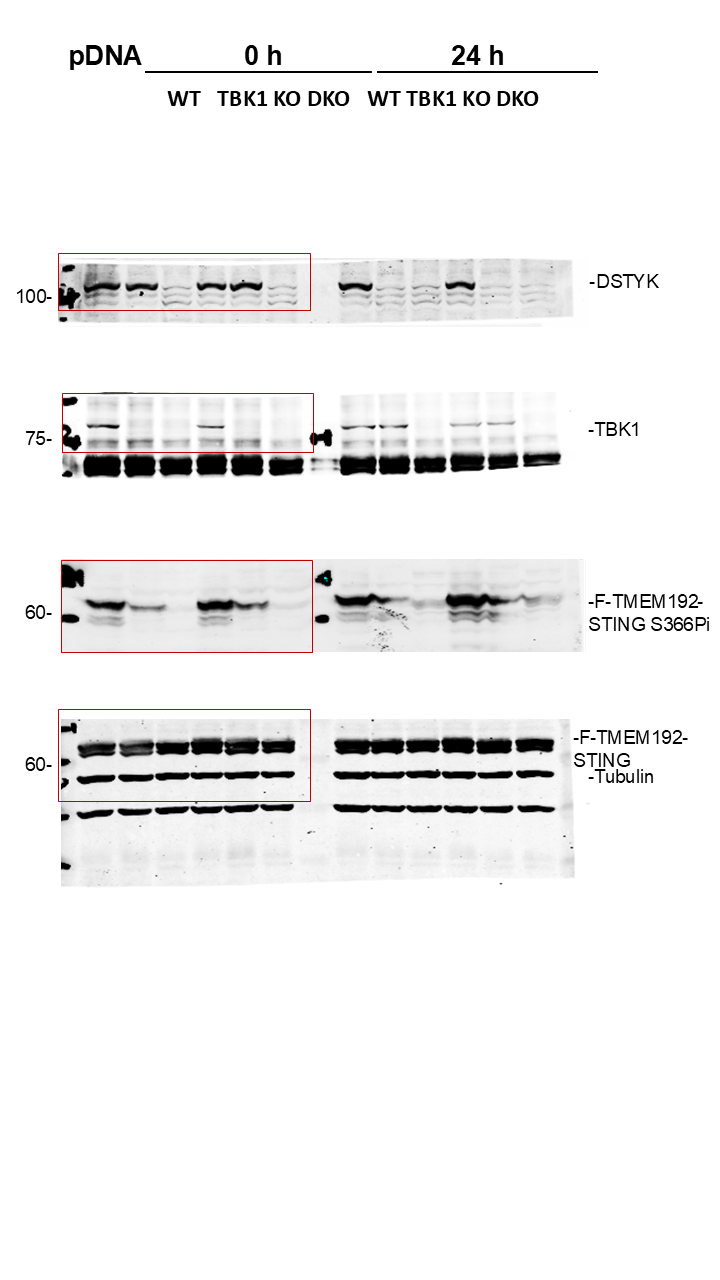

Supplement: Supplementary file 5 — Source data Fig. 4 [file 44319_2025_394_MOESM5_ESM.zip › Figure 5/Figure 5D/Figure 5D.TIF]

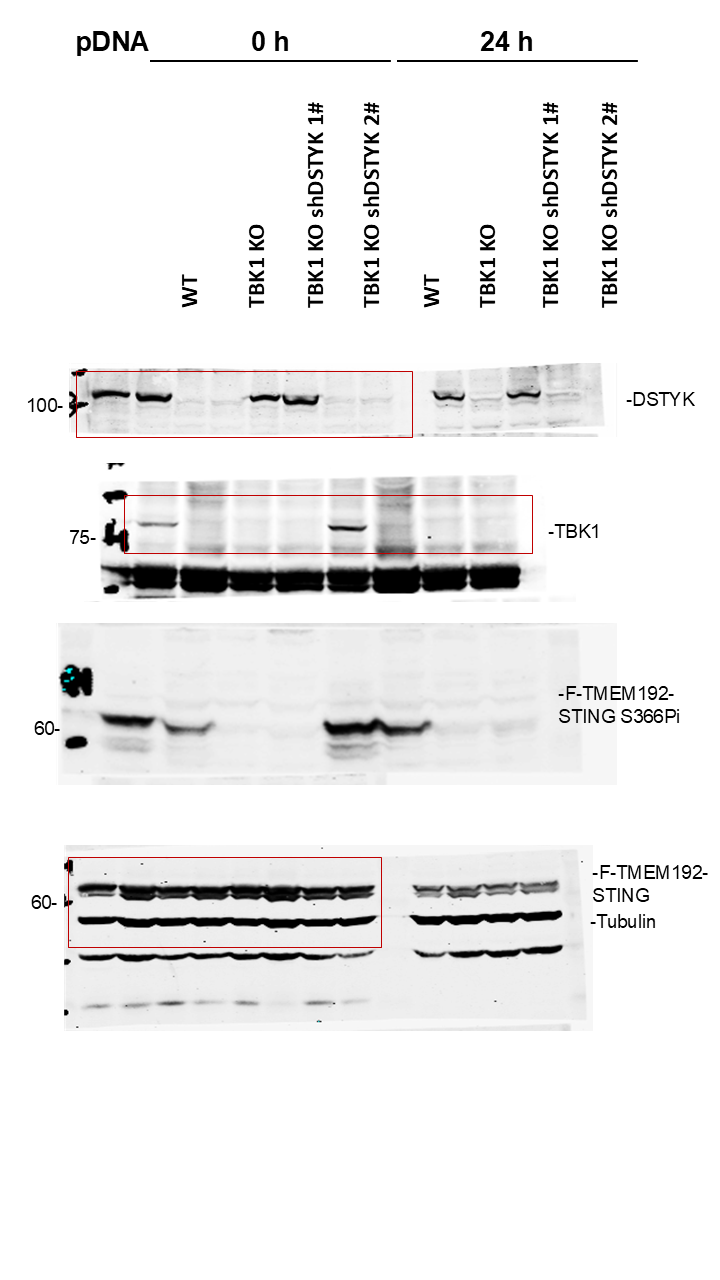

Supplement: Supplementary file 5 — Source data Fig. 4 [file 44319_2025_394_MOESM5_ESM.zip › Figure 5/Figure 5E/Figure 5E.TIF]

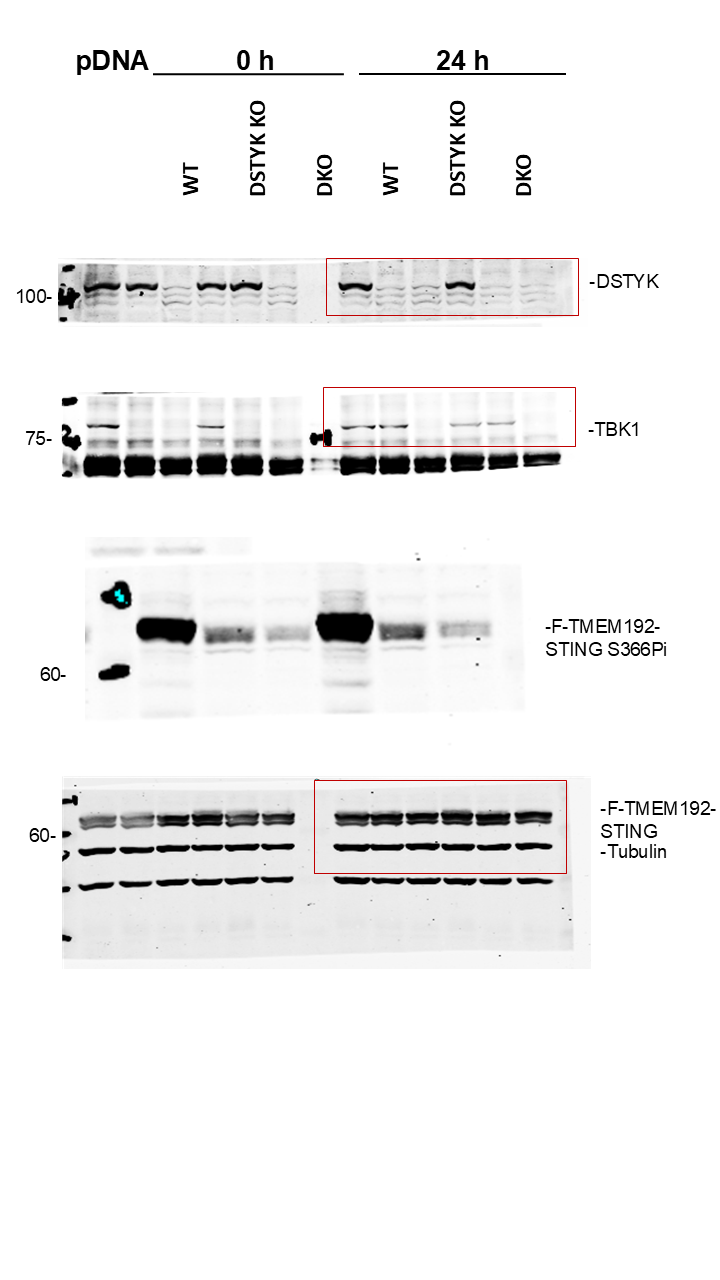

Supplement: Supplementary file 5 — Source data Fig. 4 [file 44319_2025_394_MOESM5_ESM.zip › Figure 5/Figure 5F/Figure 5F.TIF]

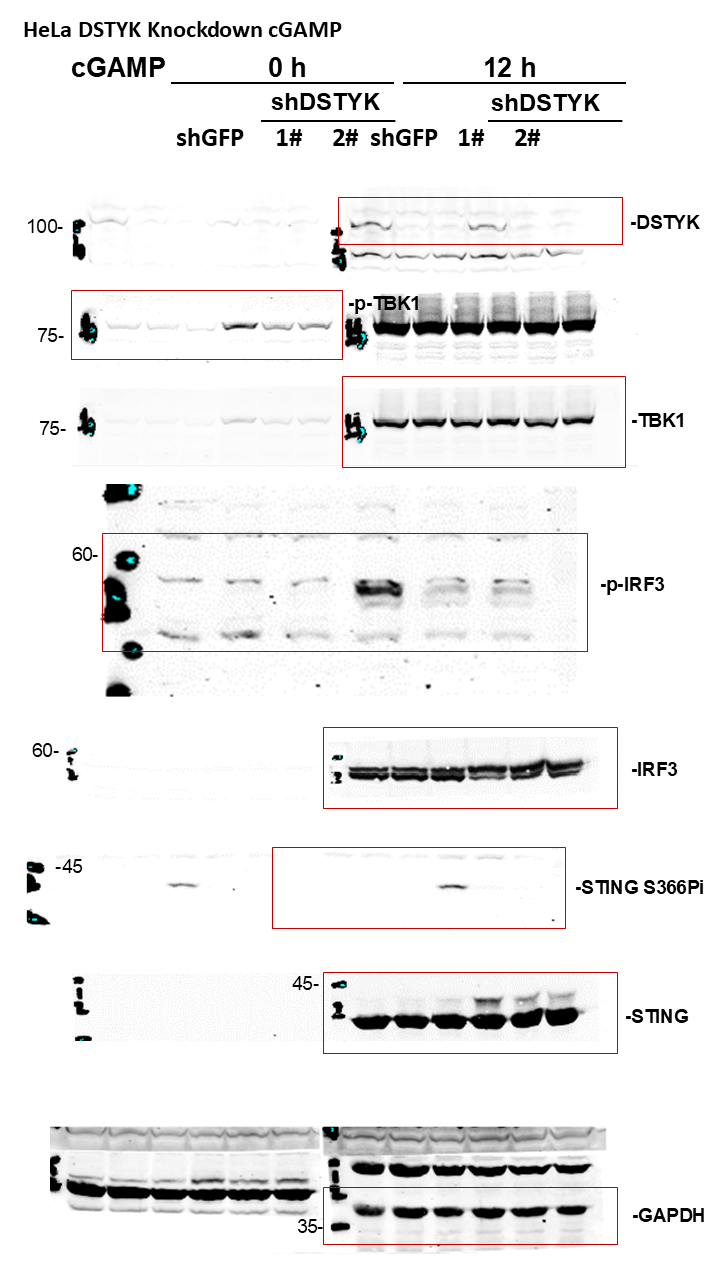

Supplement: Supplementary file 7 — Source data Fig. 6 [file 44319_2025_394_MOESM7_ESM.zip › Figure 2/Figure 2A/Figure 2A.TIF]

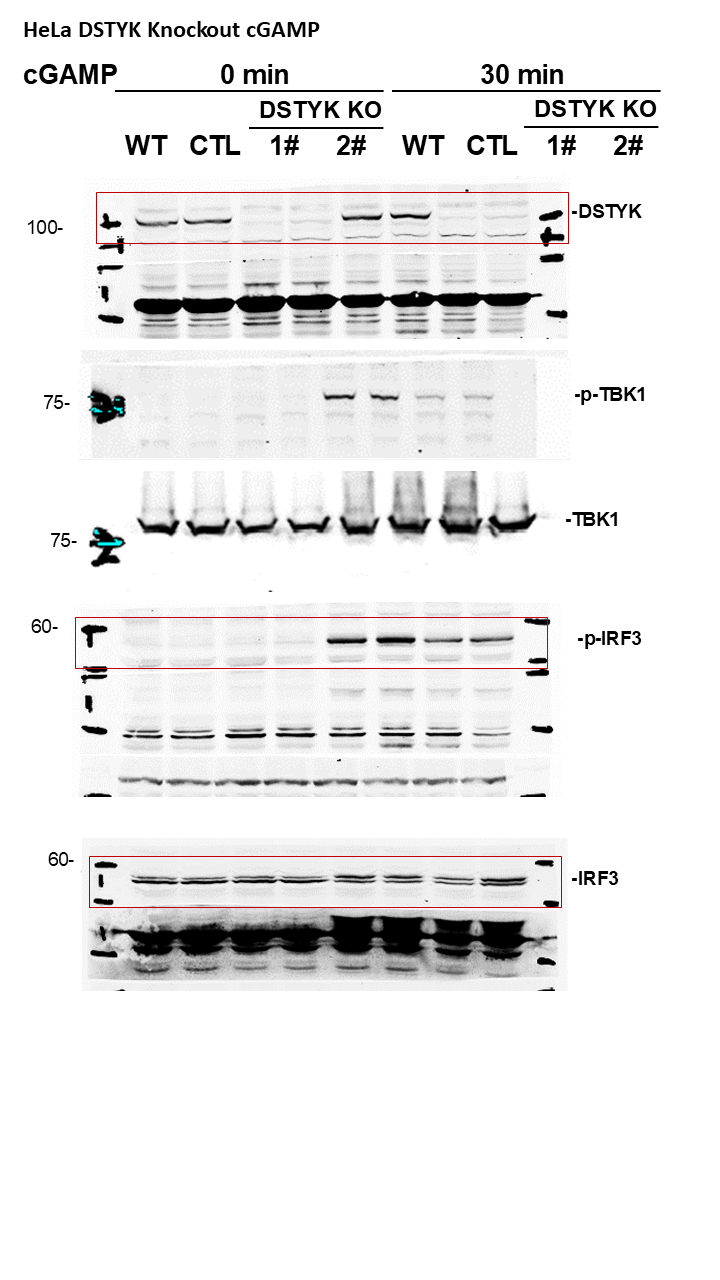

Supplement: Supplementary file 7 — Source data Fig. 6 [file 44319_2025_394_MOESM7_ESM.zip › Figure 2/Figure 2B/Figure 2B-1.TIF]

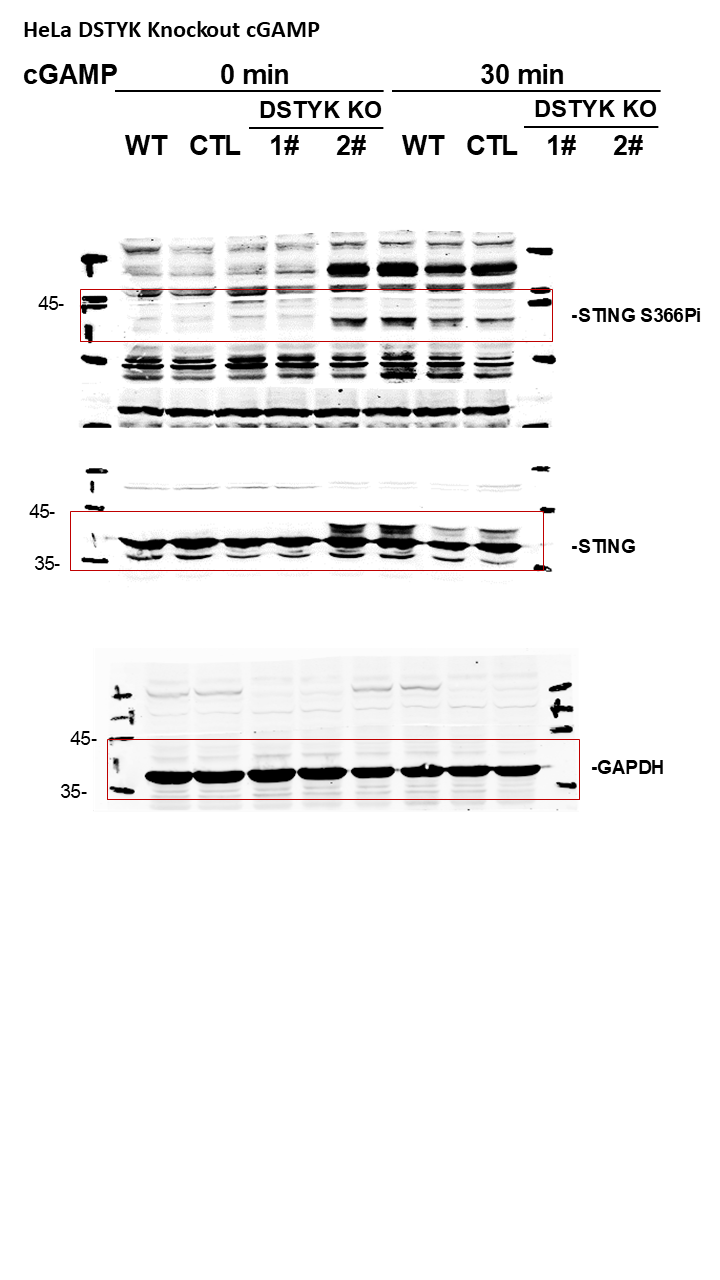

Supplement: Supplementary file 7 — Source data Fig. 6 [file 44319_2025_394_MOESM7_ESM.zip › Figure 2/Figure 2B/Figure 2B-2.TIF]

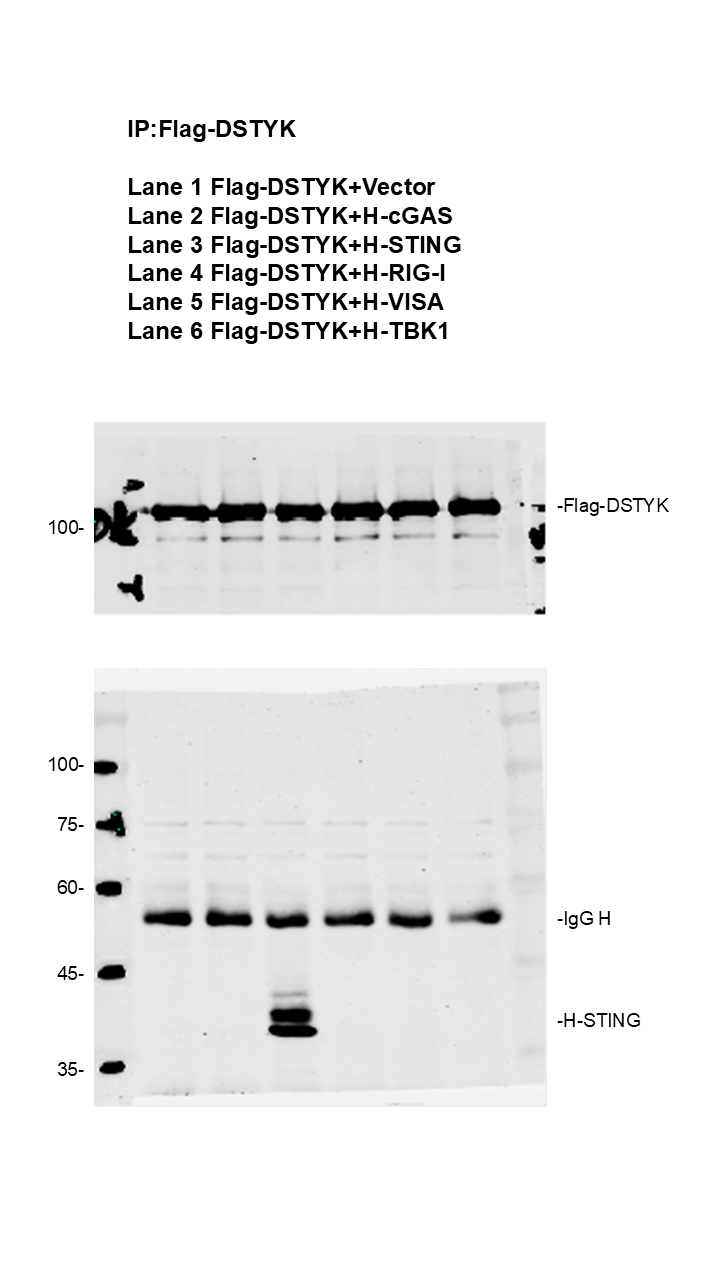

Supplement: Supplementary file 7 — Source data Fig. 6 [file 44319_2025_394_MOESM7_ESM.zip › Figure 2/Figure 2C/Figure 2C-1.TIF]

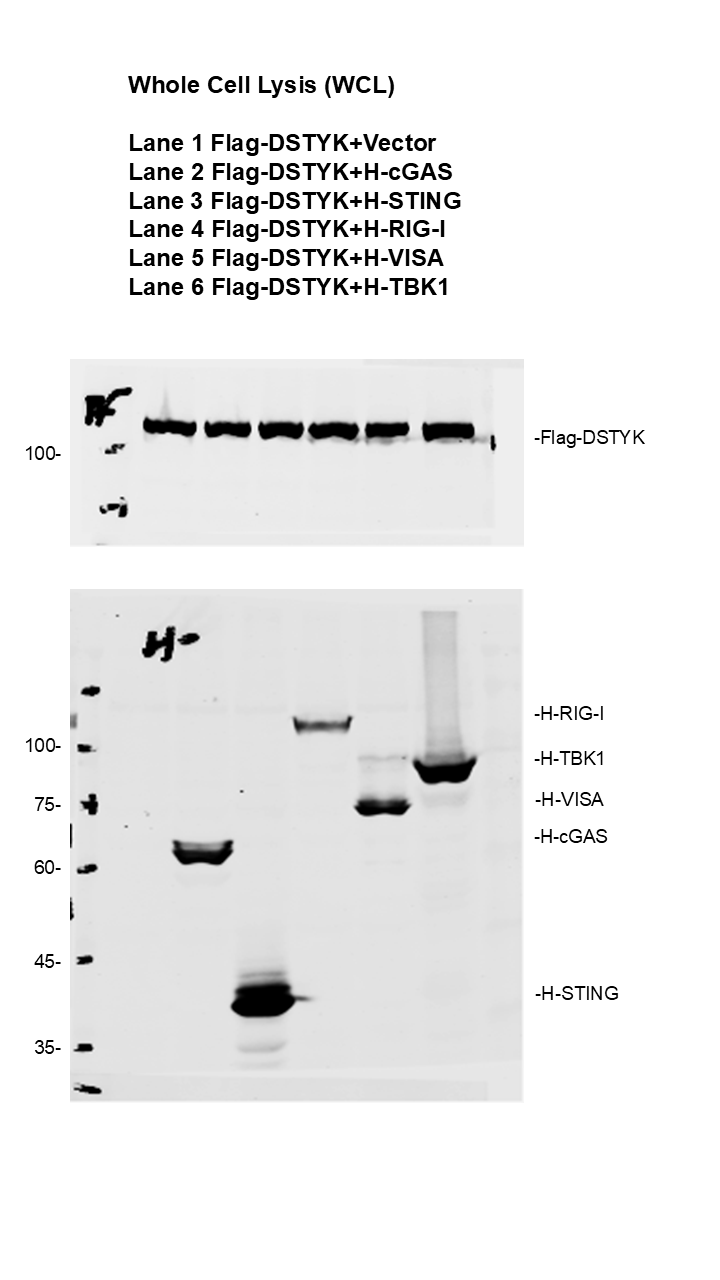

Supplement: Supplementary file 7 — Source data Fig. 6 [file 44319_2025_394_MOESM7_ESM.zip › Figure 2/Figure 2C/Figure 2C-2.TIF]

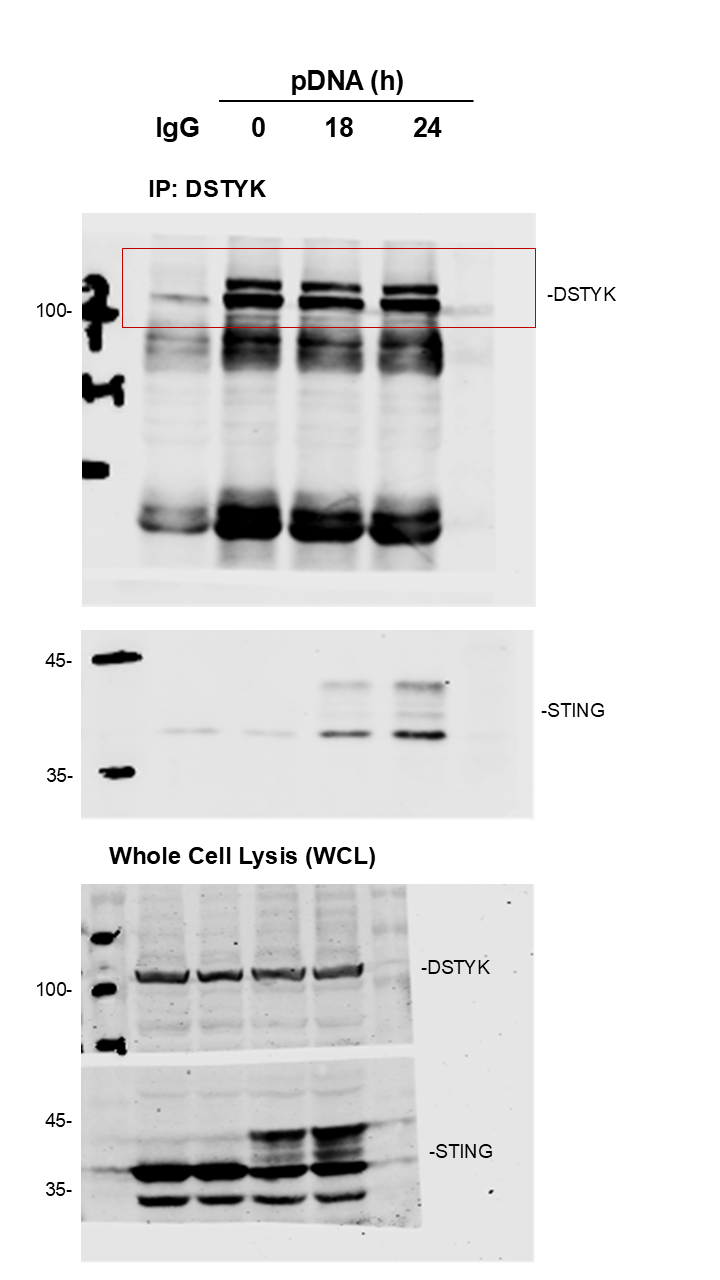

Supplement: Supplementary file 7 — Source data Fig. 6 [file 44319_2025_394_MOESM7_ESM.zip › Figure 2/Figure 2D/Figure 2D.TIF]

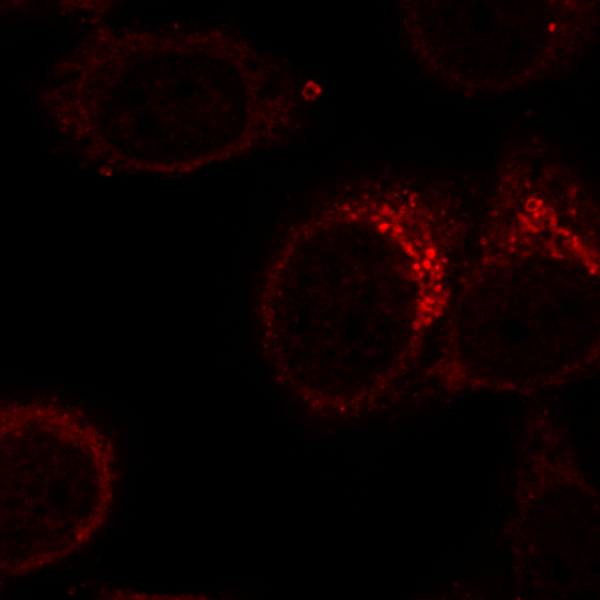

Supplement: Supplementary file 7 — Source data Fig. 6 [file 44319_2025_394_MOESM7_ESM.zip › Figure 2/Figure 2J/12 h/STING F-DK RAB7 12-1_w3CSU Cy5.TIF]

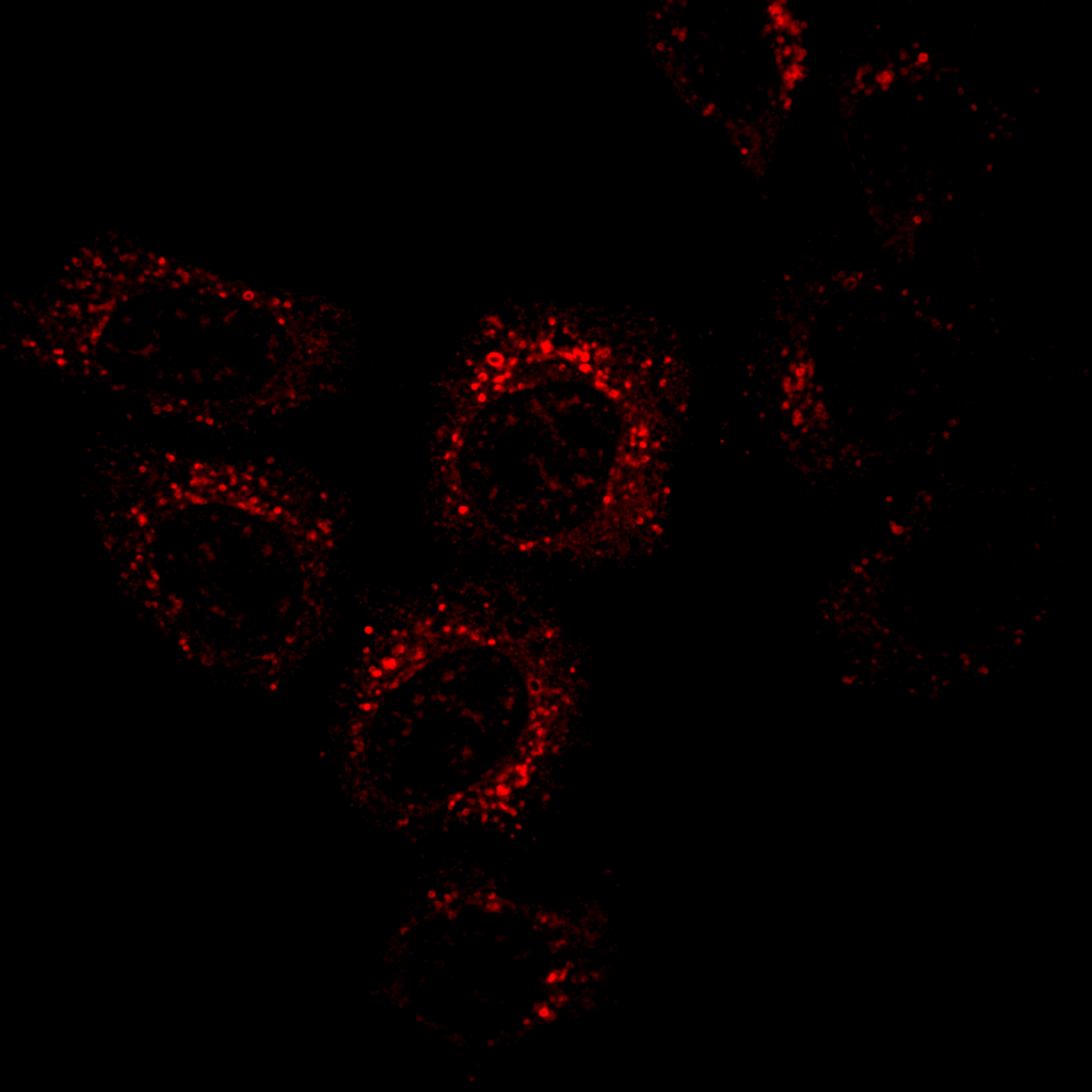

Supplement: Supplementary file 7 — Source data Fig. 6 [file 44319_2025_394_MOESM7_ESM.zip › Figure 2/Figure 2K/0 h/F-DK STING LAMP1 0-5_w3CSU Cy5.TIF]

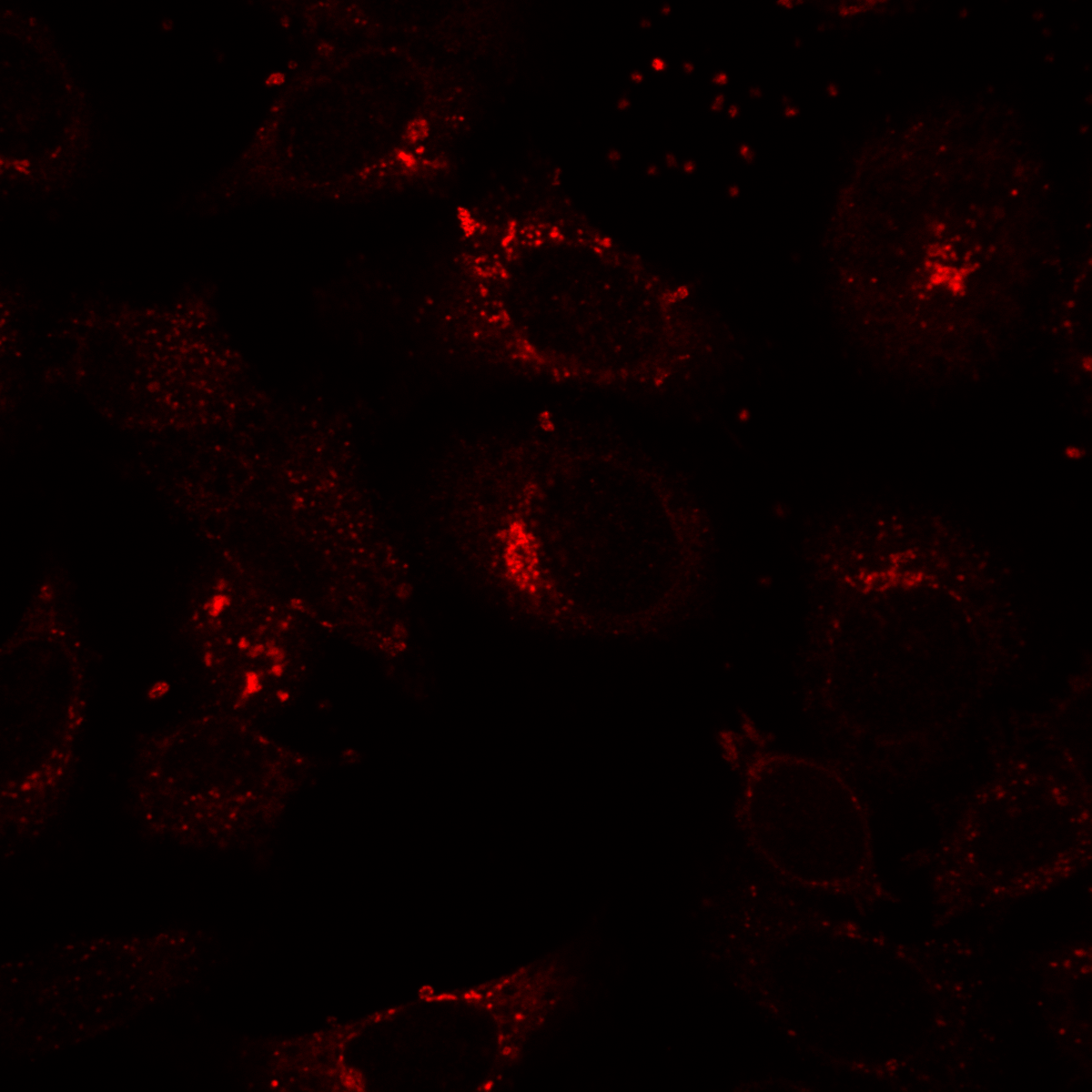

Supplement: Supplementary file 7 — Source data Fig. 6 [file 44319_2025_394_MOESM7_ESM.zip › Figure 2/Figure 2K/8 h/STING F-DK LAMP1 8-7_w3CSU Cy5.TIF]

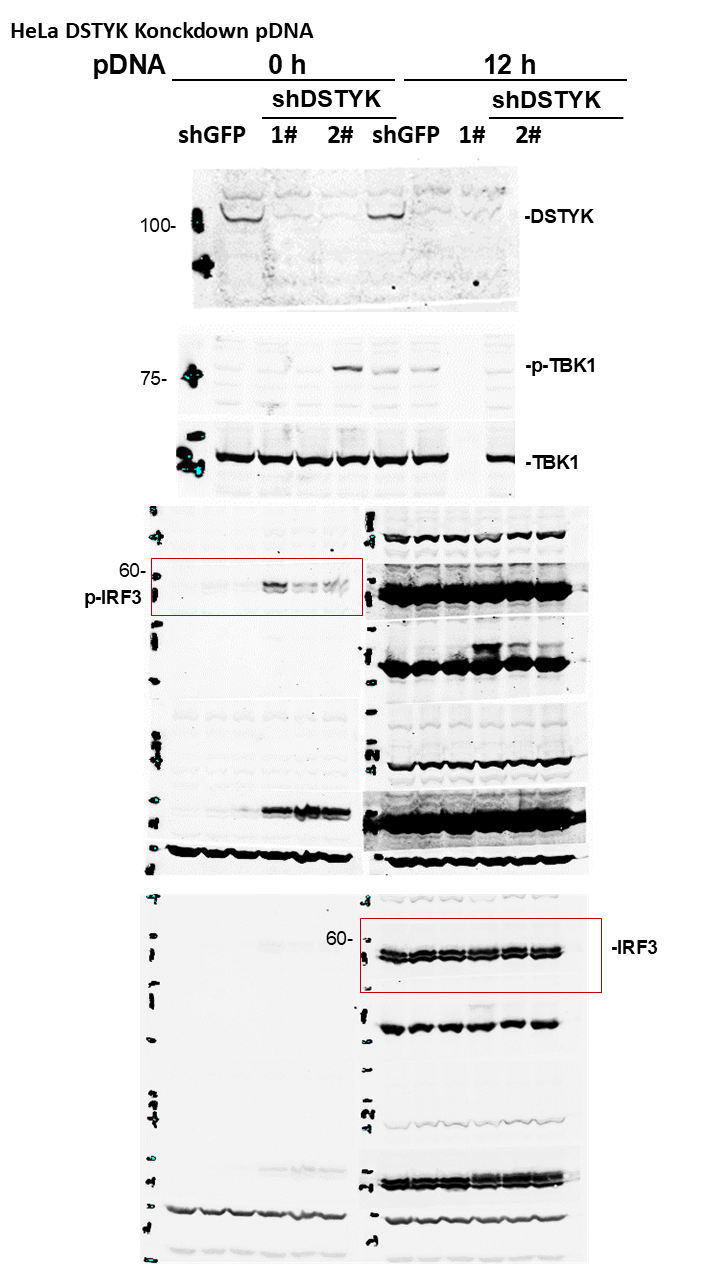

Supplement: Supplementary file 8 — EV Figures Source Data [file 44319_2025_394_MOESM8_ESM.zip › Figure EV3/Figure EV3A/Figure EV2A-1.TIF]

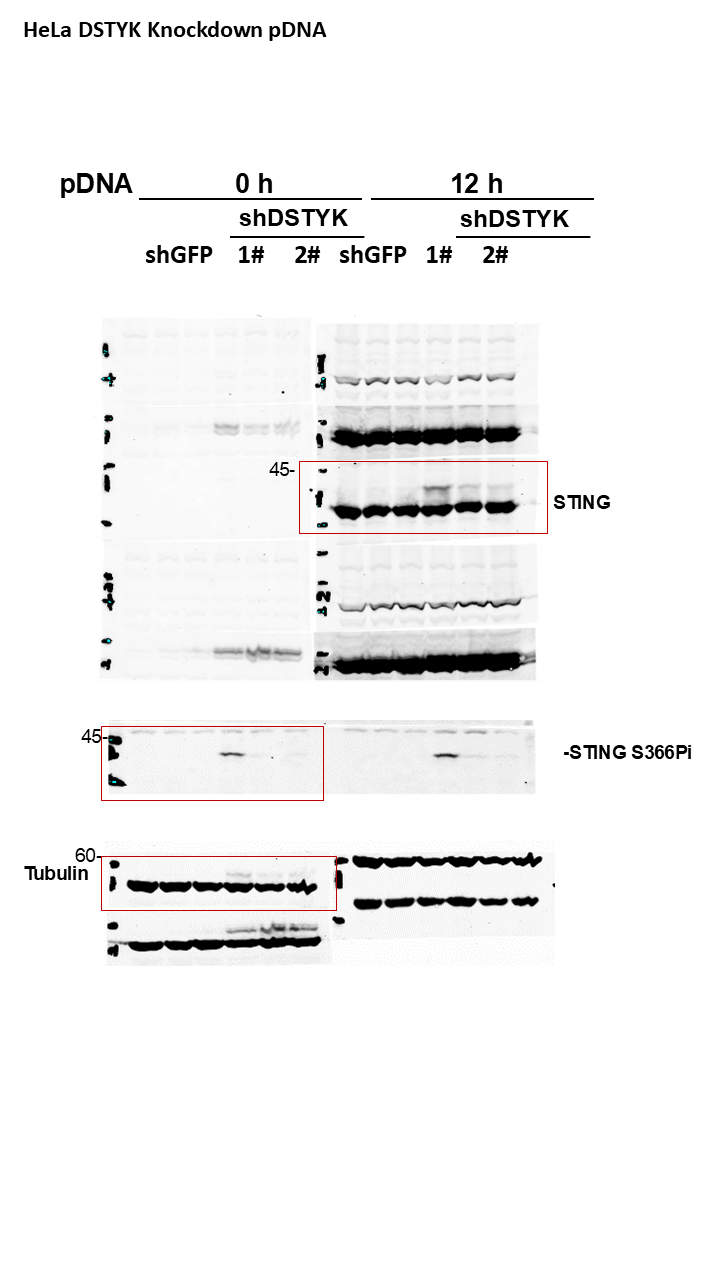

Supplement: Supplementary file 8 — EV Figures Source Data [file 44319_2025_394_MOESM8_ESM.zip › Figure EV3/Figure EV3A/Figure EV2A-2.TIF]

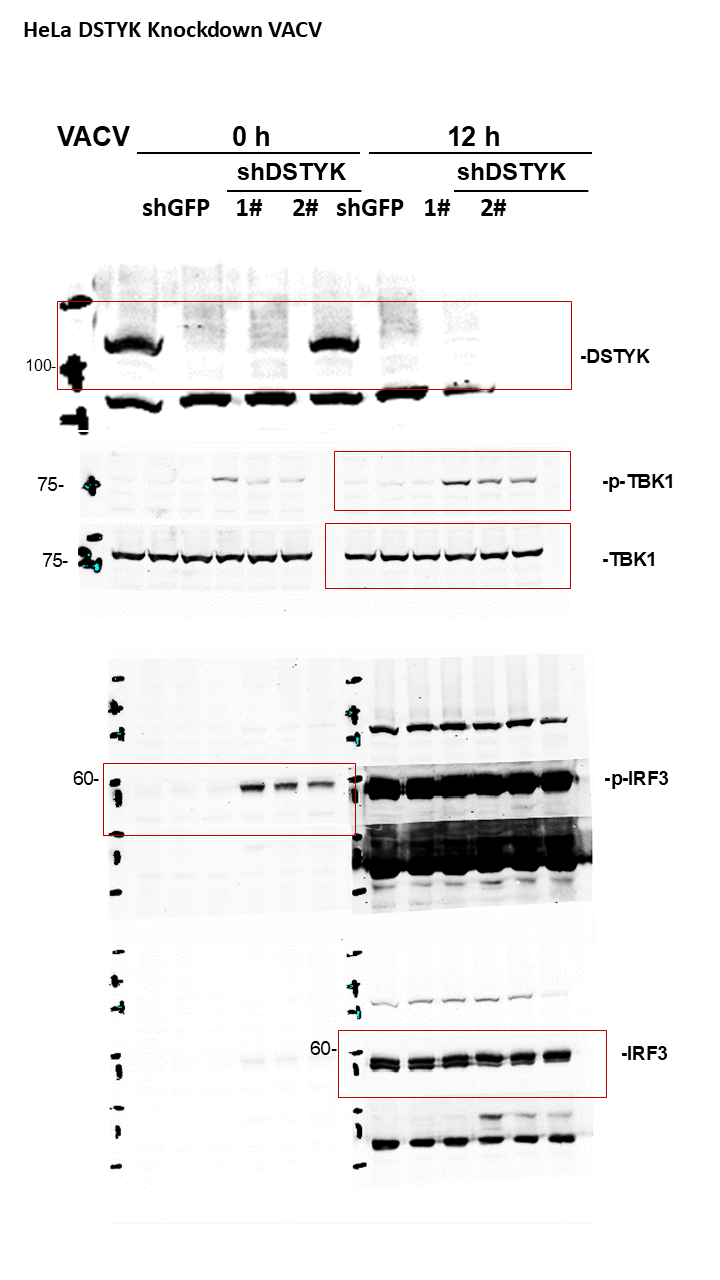

Supplement: Supplementary file 8 — EV Figures Source Data [file 44319_2025_394_MOESM8_ESM.zip › Figure EV3/Figure EV3B/Figure EV2B-1.TIF]

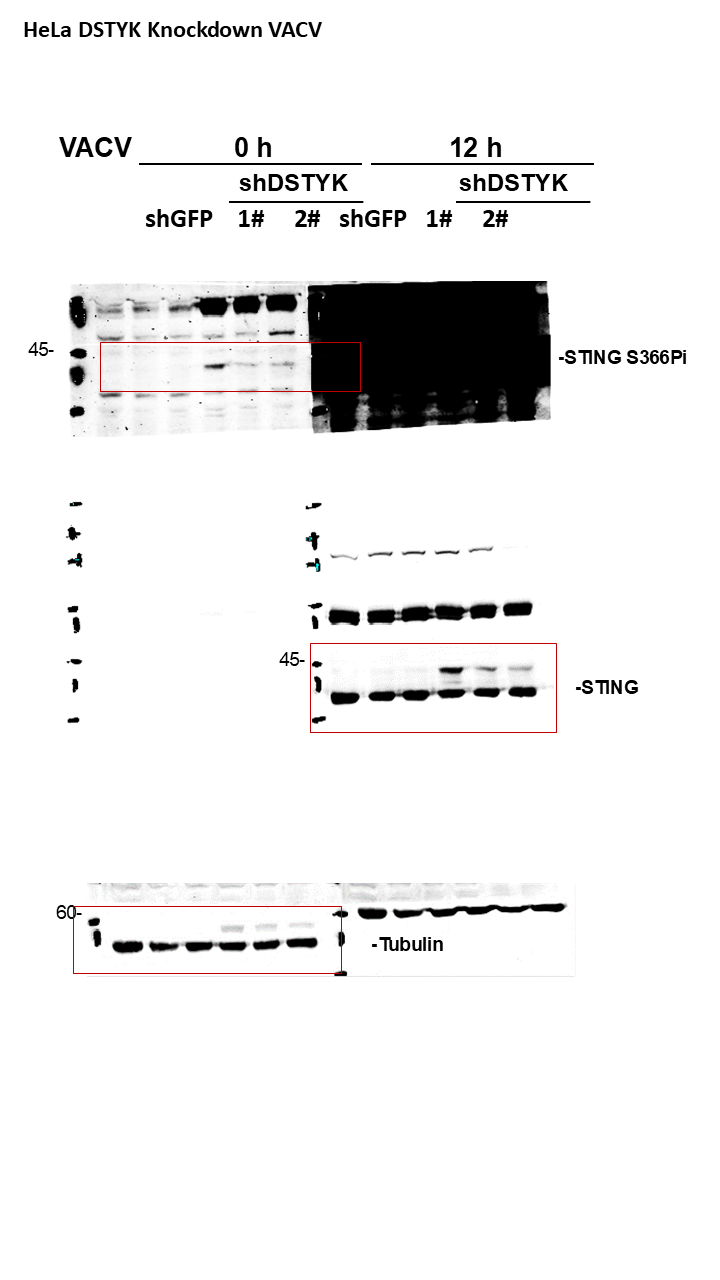

Supplement: Supplementary file 8 — EV Figures Source Data [file 44319_2025_394_MOESM8_ESM.zip › Figure EV3/Figure EV3B/Figure EV2B-2.TIF]

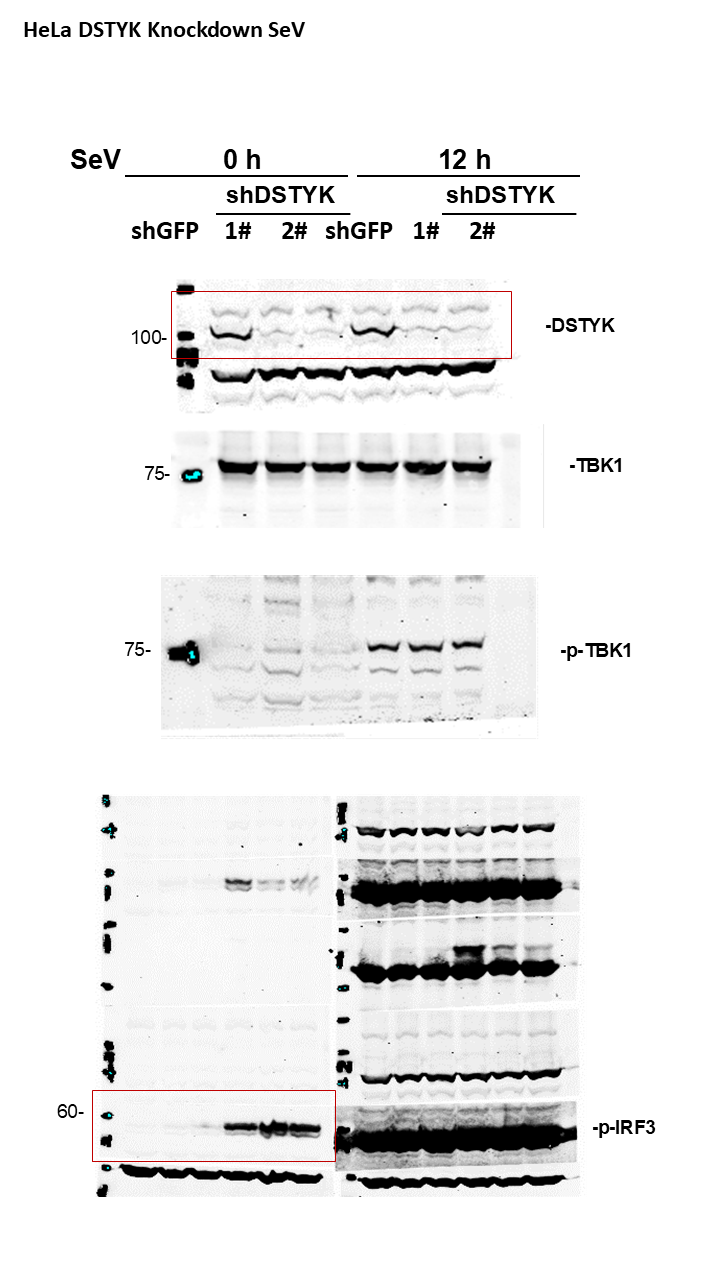

Supplement: Supplementary file 8 — EV Figures Source Data [file 44319_2025_394_MOESM8_ESM.zip › Figure EV3/Figure EV3C/Figure EV2C-1.TIF]

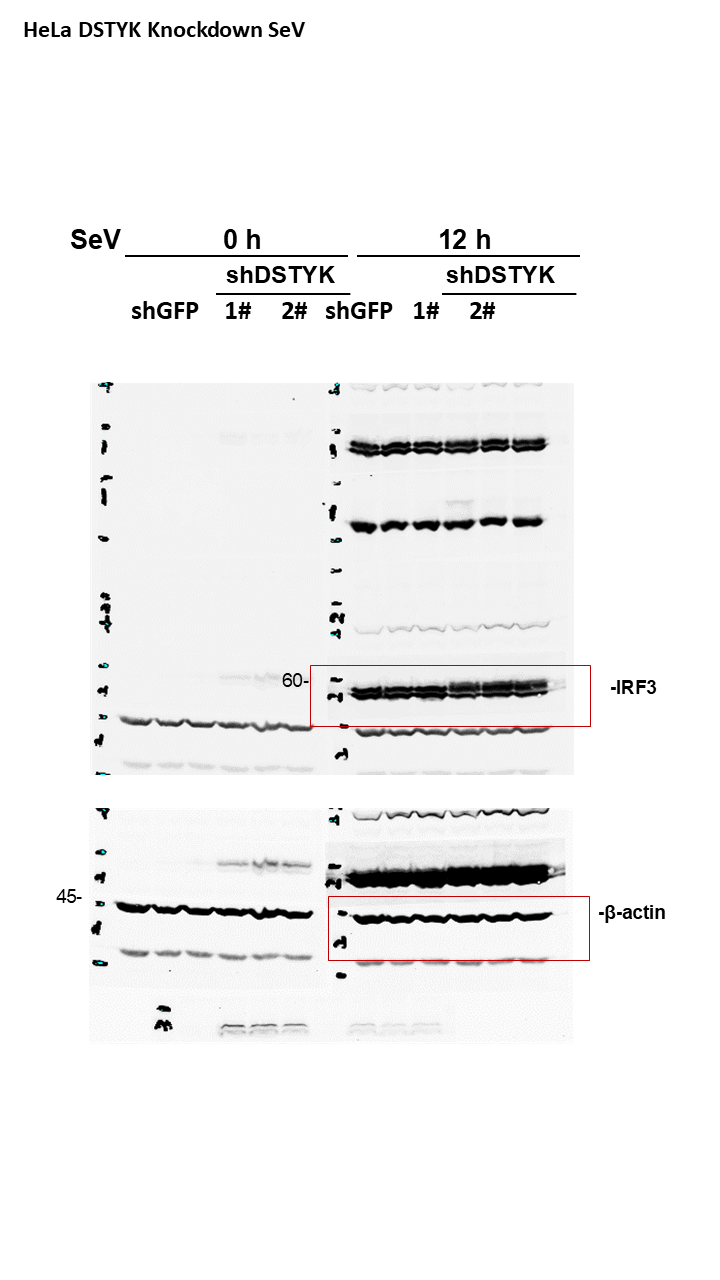

Supplement: Supplementary file 8 — EV Figures Source Data [file 44319_2025_394_MOESM8_ESM.zip › Figure EV3/Figure EV3C/Figure EV2C-2.TIF]
