# Supplementary material for: DSTYK phosphorylates STING at late endosomes to promote STING signaling
Source: EMBO Rep. 2025 Feb 20;26(6):1620–46. doi: 10.1038/s44319-025-00394-9 (PMC11933320; doi:10.1038/s44319-025-00394-9)
Supplement: Supplementary file 9 — Expanded View Figures [file 44319_2025_394_MOESM9_ESM.pdf]

Expanded View Figures

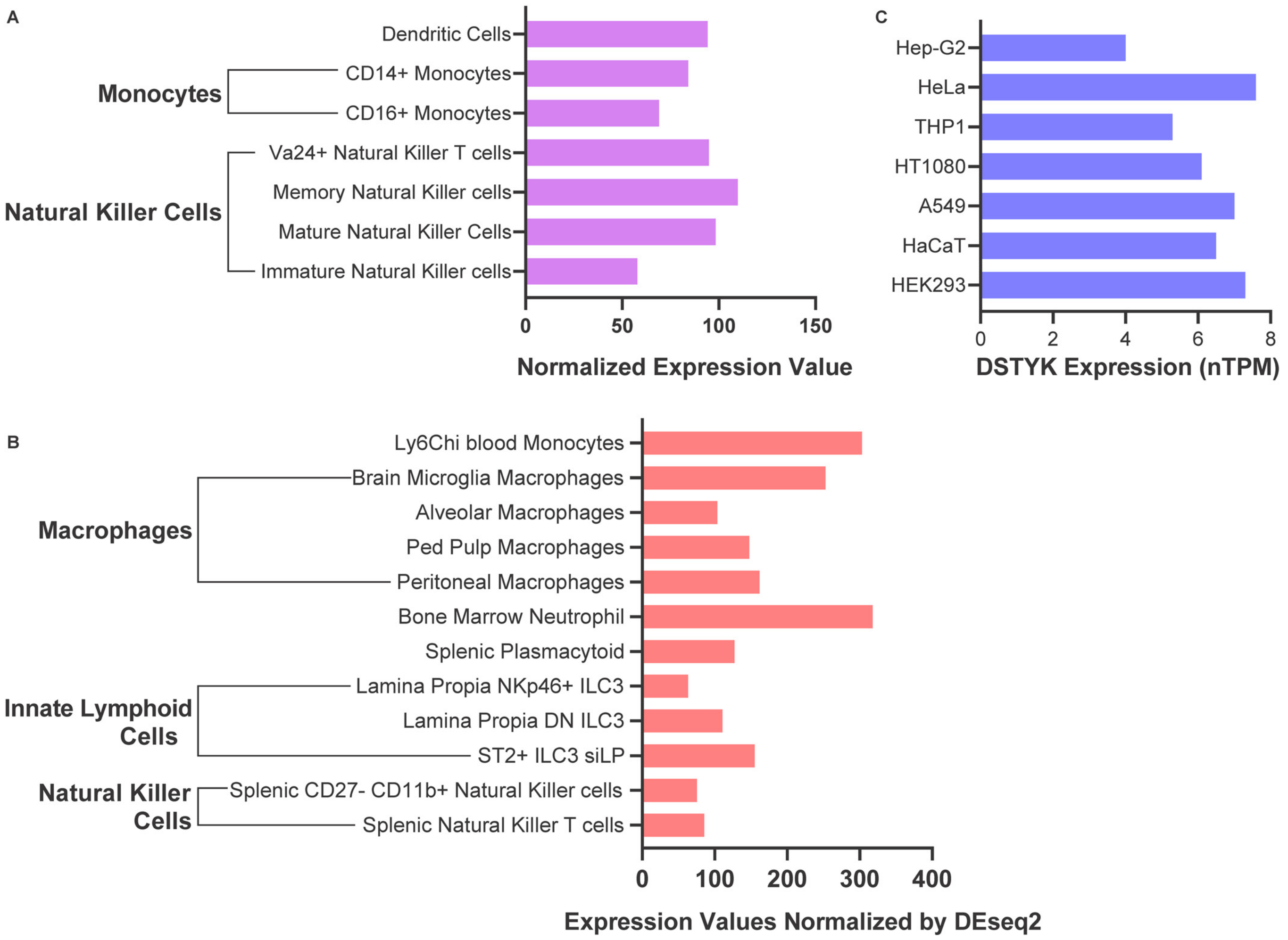

Figure EV1. DSTYK is widely expressed among primary immune cells and human cell lines.

Analysis of the expression profile of human DSTYK (**A**) and mouse Dstyk (**B**) in primary innate immune cells are derived from Gene Skyline in ImmGen, including natural killer cells, innate lymphoid cells, neutrophils, dendritic cells, and macrophages. Analysis of the expression profile of human DSTYK in common human cell lines is derived from The Human Protein Atlas (**C**), including HEK293, HaCaT, A549, HT1080, THP1, HeLa, and Hep-G2.

© The Author(s)

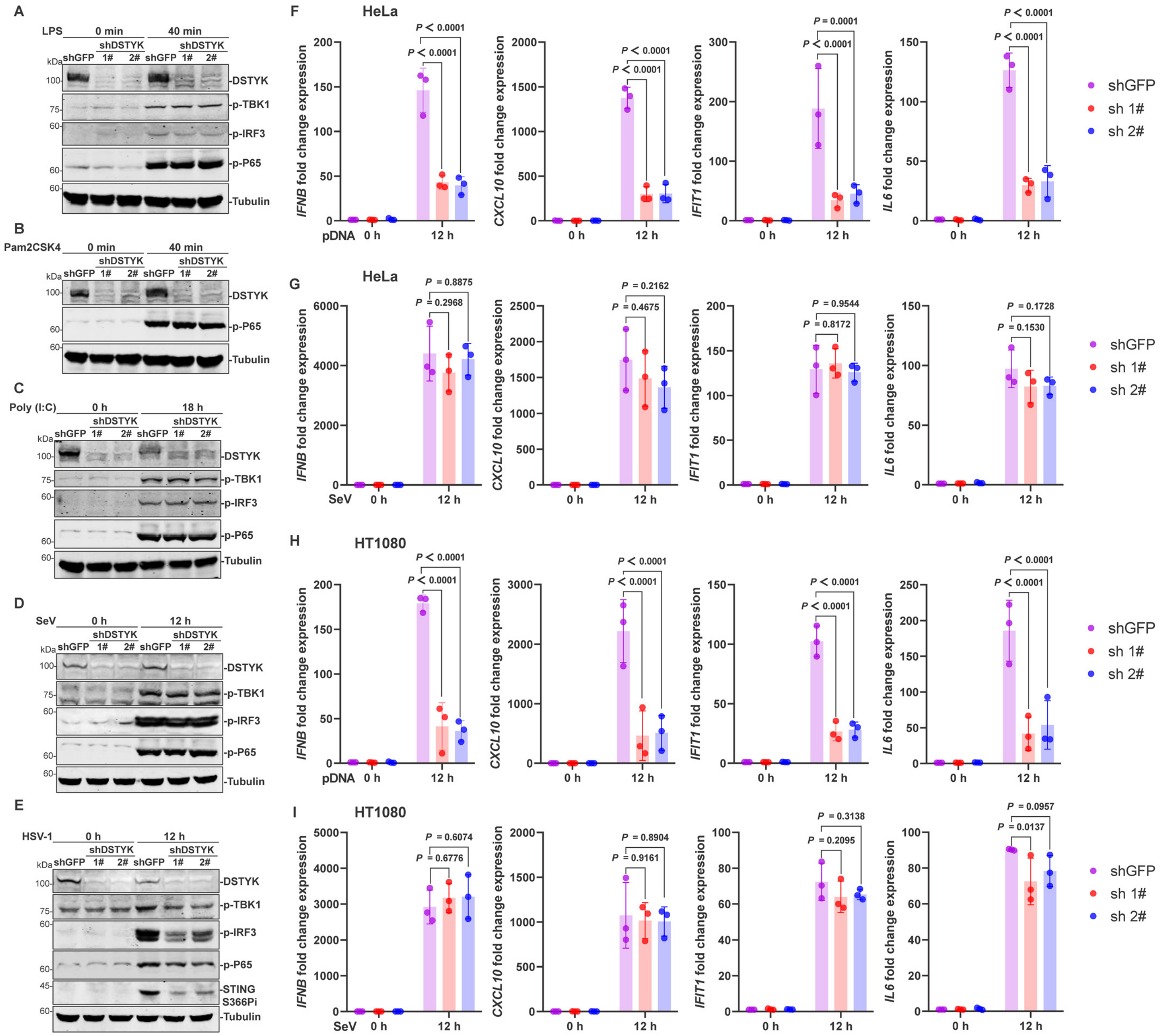

Figure EV2. DSTYK deficiency inhibits the cytosolic DNA-triggered cGAS–STING pathway.

(**A–E**) *DSTYK*-knockdown THP1-derived macrophages and control cells (shGFP) were stimulated with LPS (1 µg/mL, **A**), Pam2CSK4 (1 ng/mL, **B**), poly (I:C) (10 µg/mL, **C**), SeV (**D**) and HSV-1 (**E**) for the indicated amounts of time, and phosphorylation of TBK1, IRF3, P65 and STING at Ser366 was detected by western blotting. (**F–I**) *DSTYK* knockdown in HeLa and HT1080 cells inhibits cytosolic DNA-triggered production of *IFNB*, ISGs and genes encoding proinflammatory cytokines. *DSTYK*-knockdown HeLa (**F**, **G**) and HT1080 (**H**, **I**) cells and control cells (shGFP) were stimulated with pDNA transfection (1 µg/mL; **F**, **H**) and SeV (**G**, **I**) for 12 h, and *IFNB*, ISG (*CXCL10* and *IFIT1*) and proinflammatory cytokine (*IL6*) production was detected by quantitative PCR ($n = 3$ biological replicates). Data are representative of at least three independent experiments. Data in (**F–I**) are shown as the mean ± SD. *P* values were determined by two-way ANOVA and the exact *P* values are shown in the figures. Source data are available online for this figure.

 © The Author(s)

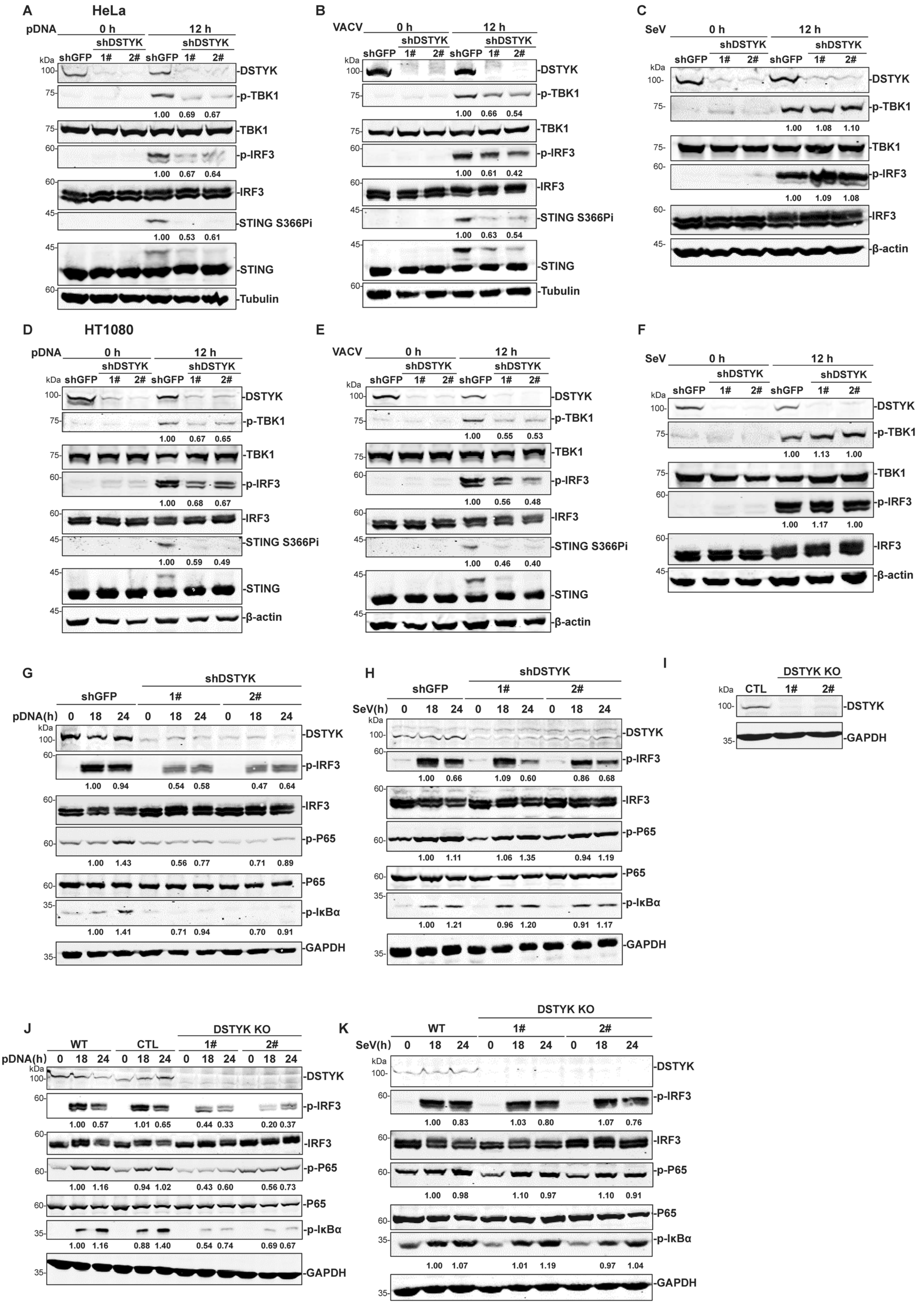
A HeLa
pDNA 0 h 12 h
shDSTYK shDSTYK
kDa shGFP 1# 2# shGFP 1# 2#
-DSTYK
-p-TBK1
1.00 0.69 0.67
-TBK1
-p-IRF3
1.00 0.67 0.64
-IRF3
-STING S366Pi
1.00 0.53 0.61
-STING
-Tubulin
B
VACV 0 h 12 h
shDSTYK shDSTYK
kDa shGFP 1# 2# shGFP 1# 2#
-DSTYK
-p-TBK1
1.00 0.66 0.54
-TBK1
-p-IRF3
1.00 0.61 0.42
-IRF3
-STING S366Pi
1.00 0.63 0.54
-STING
-Tubulin
C
SeV 0 h 12 h
shDSTYK shDSTYK
kDa shGFP 1# 2# shGFP 1# 2#
-DSTYK
-p-TBK1
1.00 1.08 1.10
-TBK1
-p-IRF3
1.00 1.09 1.08
-IRF3
-β-actin
D HT1080
pDNA 0 h 12 h
shDSTYK shDSTYK
kDa shGFP 1# 2# shGFP 1# 2#
-DSTYK
-p-TBK1
1.00 0.67 0.65
-TBK1
-p-IRF3
1.00 0.68 0.67
-IRF3
-STING S366Pi
1.00 0.59 0.49
-STING
-β-actin
E
VACV 0 h 12 h
shDSTYK shDSTYK
kDa shGFP 1# 2# shGFP 1# 2#
-DSTYK
-p-TBK1
1.00 0.55 0.53
-TBK1
-p-IRF3
1.00 0.56 0.48
-IRF3
-STING S366Pi
1.00 0.46 0.40
-STING
-β-actin
F
SeV 0 h 12 h
shDSTYK shDSTYK
kDa shGFP 1# 2# shGFP 1# 2#
-DSTYK
-p-TBK1
1.00 1.13 1.00
-TBK1
-p-IRF3
1.00 1.17 1.00
-IRF3
-β-actin
G
shDSTYK
shGFP 1# 2#
pDNA(h) 0 18 24 0 18 24 0 18 24
-DSTYK
-p-IRF3
1.00 0.94 0.54 0.58 0.47 0.64
-IRF3
-p-P65
1.00 1.43 0.56 0.77 0.71 0.89
-P65
-p-IκBα
1.00 1.41 0.71 0.94 0.70 0.91
-GAPDH
H
shDSTYK
shGFP 1# 2#
SeV(h) 0 18 24 0 18 24 0 18 24
-DSTYK
-p-IRF3
1.00 0.66 1.09 0.60 0.86 0.68
-IRF3
-p-P65
1.00 1.11 1.06 1.35 0.94 1.19
-P65
-p-IκBα
1.00 1.21 0.96 1.20 0.91 1.17
-GAPDH
I
DSTYK KO
kDa CTL 1# 2#
-DSTYK
-GAPDH
J
DSTYK KO
WT CTL 1# 2#
pDNA(h) 0 18 24 0 18 24 0 18 24 0 18 24
-DSTYK
-p-IRF3
1.00 0.57 1.01 0.65 0.44 0.33 0.20 0.37
-IRF3
-p-P65
1.00 1.16 0.94 1.02 0.43 0.60 0.56 0.73
-P65
-p-IκBα
1.00 1.16 0.88 1.40 0.54 0.74 0.69 0.67
-GAPDH
K
DSTYK KO
WT 1# 2#
SeV(h) 0 18 24 0 18 24 0 18 24
-DSTYK
-p-IRF3
1.00 0.83 1.03 0.80 1.07 0.76
-IRF3
-p-P65
1.00 0.98 1.10 0.97 1.10 0.91
-P65
-p-IκBα
1.00 1.07 1.01 1.19 0.97 1.04
-GAPDH

Figure EV3. DSTYK deficiency inhibits the cytosolic DNA-triggered phosphorylation of TBK1, IRF3, STING, P65, and IκBα.

(**A–F**) *DSTYK* knockdown in HeLa and HT1080 cells inhibits cytosolic DNA-triggered phosphorylation of TBK1, IRF3, and STING at Ser366. *DSTYK*-knockdown HeLa (**A–C**) and HT1080 (**D–F**) cells and control cells (shGFP) were stimulated with pDNA transfection (1 μg/mL; **A**, **D**), VACV (**B**, **E**) and SeV (**C**, **F**) for 12 h, and phosphorylation of TBK1, IRF3 and STING at Ser366 was detected by western blotting. (**G**, **H**) *DSTYK* knockdown in HeLa cells inhibits cytosolic DNA-triggered phosphorylation of IRF3, P65, and IκBα. *DSTYK*-knockdown HeLa cells and control cells (shGFP) were stimulated with pDNA transfection (1 μg/mL; **G**) and SeV (**H**) for the indicated amounts of time, and phosphorylation of IRF3, P65, and IκBα was detected by western blotting. (**I**) Detection of *DSTYK* knockout by western blotting. (**J**, **K**) *DSTYK* knockout in HeLa cells inhibits cytosolic DNA-triggered phosphorylation of IRF3, P65, and IκBα. *DSTYK*-knockout HeLa cells and control cells (WT and CTL) were stimulated with pDNA transfection (1 μg/mL; **J**) and SeV (**K**) for the indicated amounts of time, and phosphorylation of IRF3, P65, and IκBα was detected by western blotting. Data are representative of at least three independent experiments. Quantification of the indicated band intensities was performed in ImageJ, and quantification results are labeled below the indicated bands ($n = 3$ technical replicates). Source data are available online for this figure.

 © The Author(s)

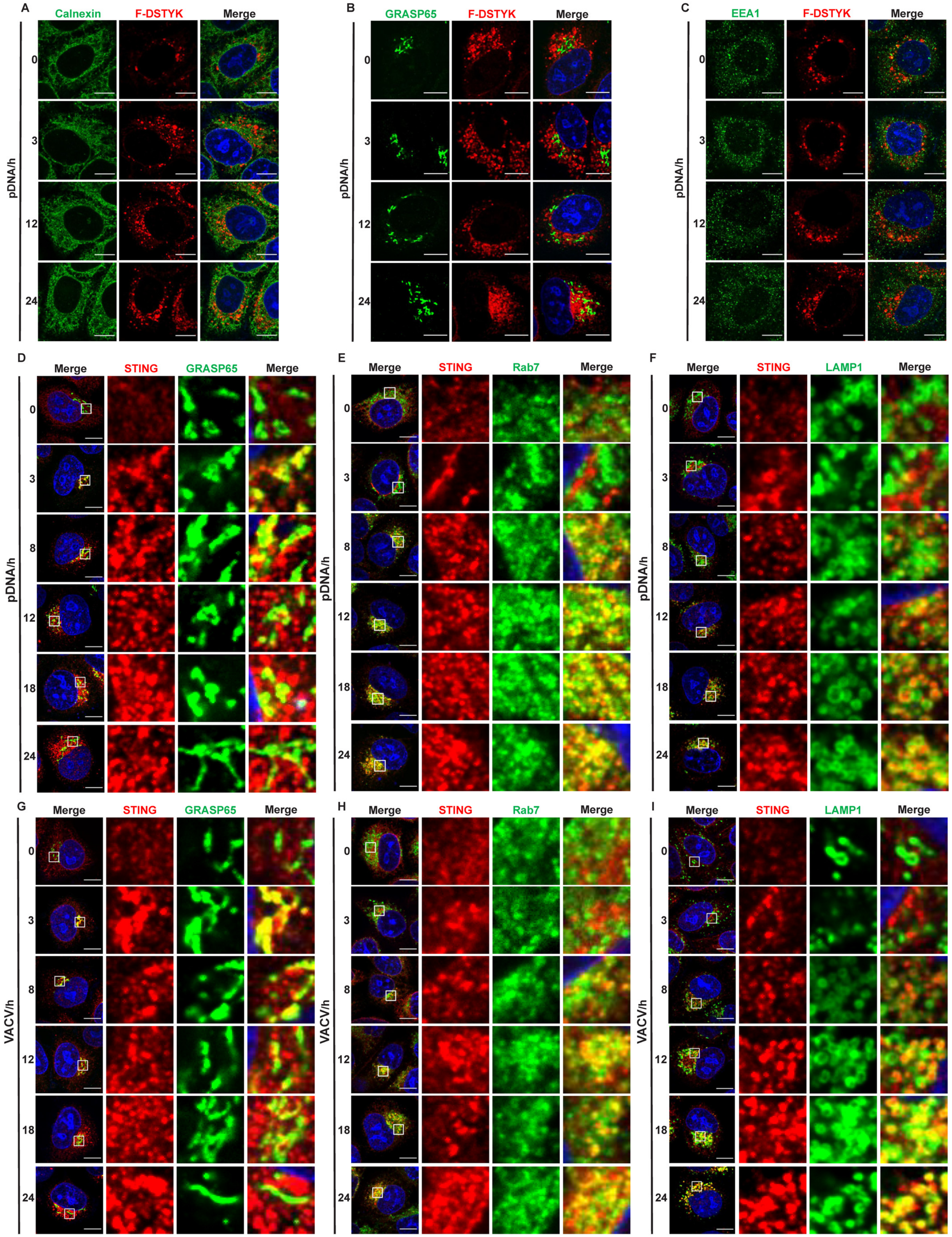
A
Calnexin
F-DSTYK
Merge
pDNA/h
0
3
12
24
B
GRASP65
F-DSTYK
Merge
C
EEA1
F-DSTYK
Merge
D
Merge
STING
GRASP65
Merge
0
3
8
12
18
24
E
Rab7
F
LAMP1
G
VACV/h
H
I

Figure EV4. DSTYK does not localize at the ER, Gogi or early endosome, and STING intracellular translocation.

(**A–C**) DSTYK does not colocalize with the ER, Golgi or early endosome. *DSTYK*-knockout HeLa cells stably expressing DSTYK were stimulated with pDNA transfection (1 µg/mL) for the indicated amounts of time, followed by observation of DSTYK colocalization with calnexin (**A**), GRASP65 (**B**) and EEA1 (**C**) via confocal imaging; scale bars, 10 µm. (**D–F**) STING intracellular translocation induced by pDNA transfection. WT HeLa cells were stimulated with pDNA transfection (1 µg/mL) for the indicated amounts of time, followed by confocal imaging to observe STING colocalization with GRASP65 (**D**), Rab7 (**E**) and LAMP1 (**F**); scale bars, 10 µm. (**G–I**) STING intracellular translocation induced by VACV. WT HeLa cells were stimulated with VACV for the indicated amounts of time, followed by confocal imaging to observe STING colocalization with GRASP65 (**G**), Rab7 (**H**), and LAMP1 (**I**); scale bars, 10 µm. Data are representative of at least three independent experiments.

 © The Author(s)

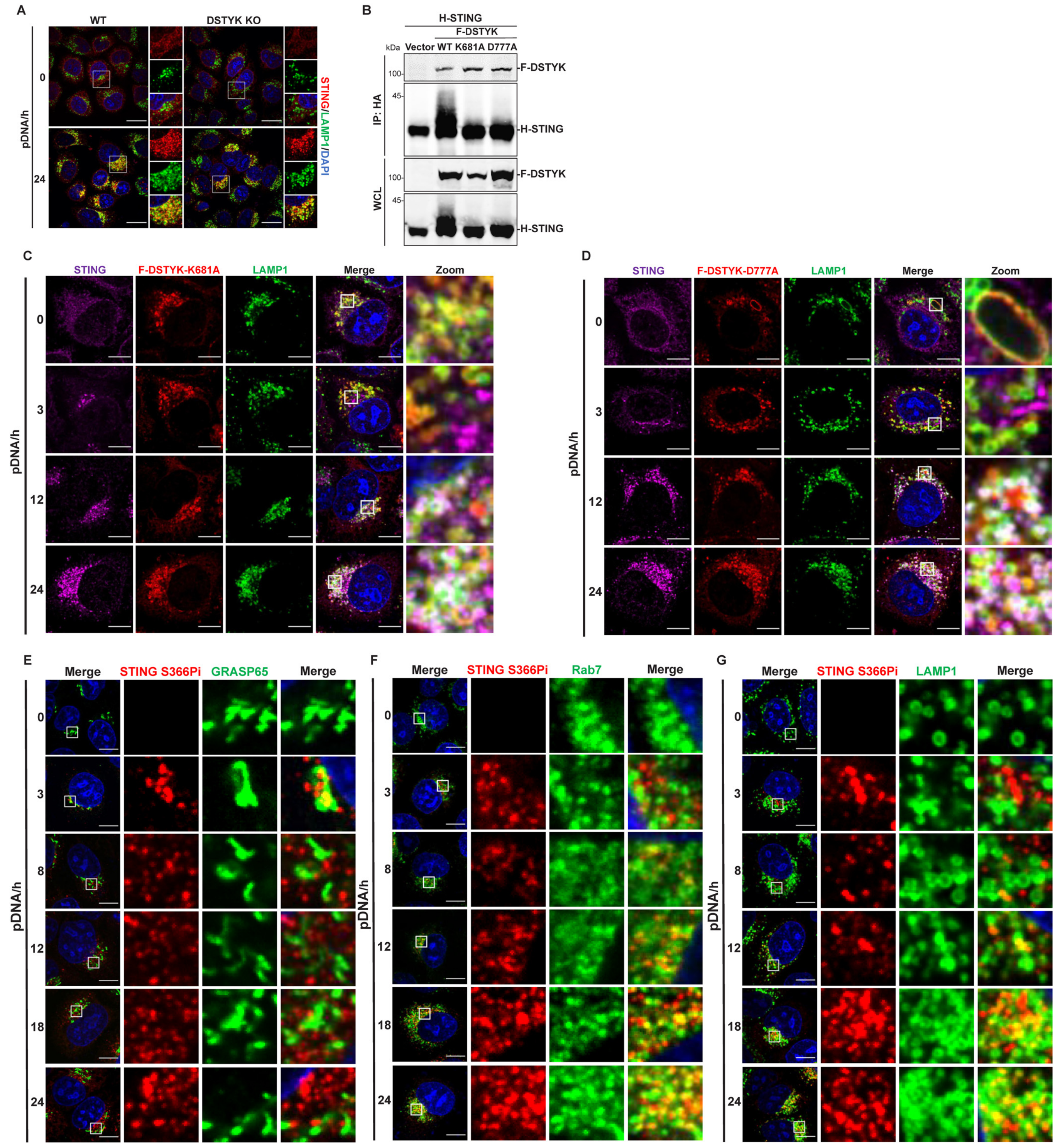
A
WT
DSTYK KO
pDNA/h
0
24
STING/LAMP1/DAPI
B
H-STING
F-DSTYK
kDa
Vector WT K681A D777A
IP: HA
WCL
100-
45-
F-DSTYK
H-STING
C
STING
F-DSTYK-K681A
LAMP1
Merge
Zoom
pDNA/h
0
3
12
24
D
STING
F-DSTYK-D777A
LAMP1
Merge
Zoom
pDNA/h
0
3
12
24
E
Merge
STING S366Pi
GRASP65
Merge
pDNA/h
0
3
8
12
18
24
F
Merge
STING S366Pi
Rab7
Merge
pDNA/h
0
3
8
12
18
24
G
Merge
STING S366Pi
LAMP1
Merge
pDNA/h
0
3
8
12
18
24

Figure EV5. DSTYK does not affect STING translocation, DSTYK kinase-dead mutants can interact with STING, and the translocation of Ser366 phosphorylated STING.

(**A**) DSTYK does not affect STING colocalization with LAMP1. *DSTYK*-knockout HeLa cells and control cells were stimulated with pDNA transfection (1 µg/mL) for 24 h, followed by confocal imaging to observe the colocalization between STING and LAMP1; scale bars, 20 µm. (**B**) DSTYK kinase-dead mutants interact with STING. HEK293T cells were transfected with the indicated plasmids (5 µg each) for 24 h. Whole-cell lysates (WCL) were examined, and cell lysates were subjected to immunoprecipitation (IP) with anti-HA, followed by immunoblotting (IB) with anti-HA and anti-Flag. (**C**, **D**) DSTYK kinase-dead mutants colocalize with STING. *DSTYK*-knockout HeLa cells stably expressing DSTYK kinase-dead mutants were stimulated with pDNA transfection (1 µg/mL) for the indicated amounts of time, followed by confocal imaging to observe DSTYK colocalization with STING and LAMP1; scale bars, 10 µm. (**E**–**G**) WT HeLa cells were stimulated with pDNA transfection (1 µg/mL) for the indicated amounts of time, followed by confocal imaging to observe phosphorylated STING colocalization with GRASP65 (**E**), Rab7 (**F**) and LAMP1 (**G**); scale bars, 10 µm. Data are representative of at least three independent experiments.

 © The Author(s)

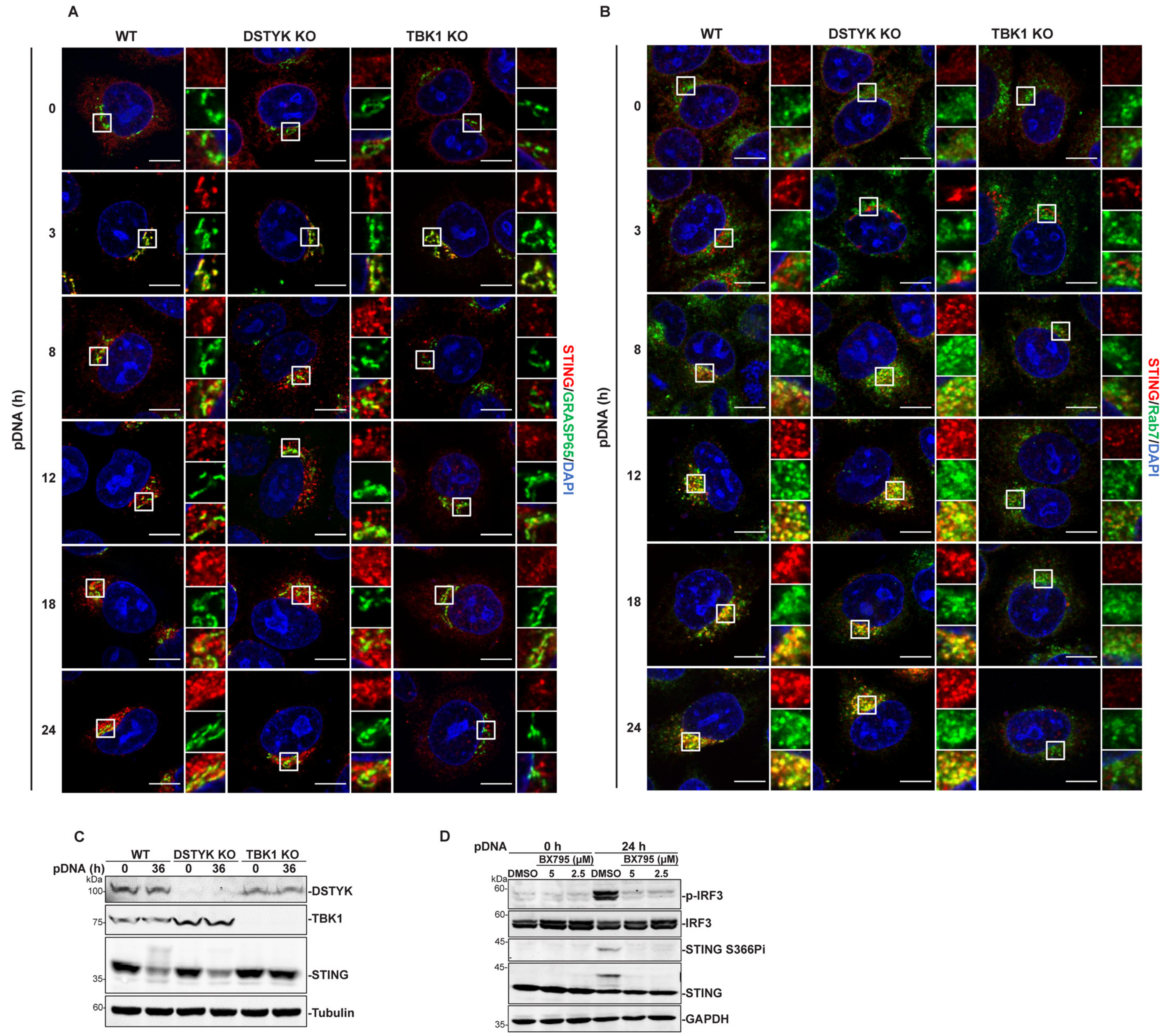

Figure EV6. ***TBK1*** **knockout inhibits STING post Golgi-trafficking, and BX795 (5 μM and 2.5 μM) inhibits the activation of STING signaling.**

(**A**, **B**) *TBK1* knockout inhibits STING post-Golgi trafficking. *TBK1*-knockout HeLa cells, *DSTYK*-knockout HeLa cells and control cells were stimulated with pDNA (1 μg/mL) for the indicated amounts of time, followed by confocal imaging to observe STING colocalization with GRAPS65 (**A**) and Rab7 (**B**); scale bars, 10 μm. (**C**) *TBK1* knockout inhibits STING degradation. *TBK1*-knockout HeLa cells, *DSTYK*-knockout HeLa cells and control cells were stimulated with pDNA (1 μg/mL) for 36 h, and STING protein level was detected via western blotting. (**D**) BX795 inhibits STING signaling. WT HeLa cells were treated with DSMO, BX795 (2.5 μM, 5 μM) for 1 h, then stimulated with pDNA for 24 h, and phosphorylation of IRF3 and STING Ser366 was detected by western blotting. Data are representative of at least three independent experiments.

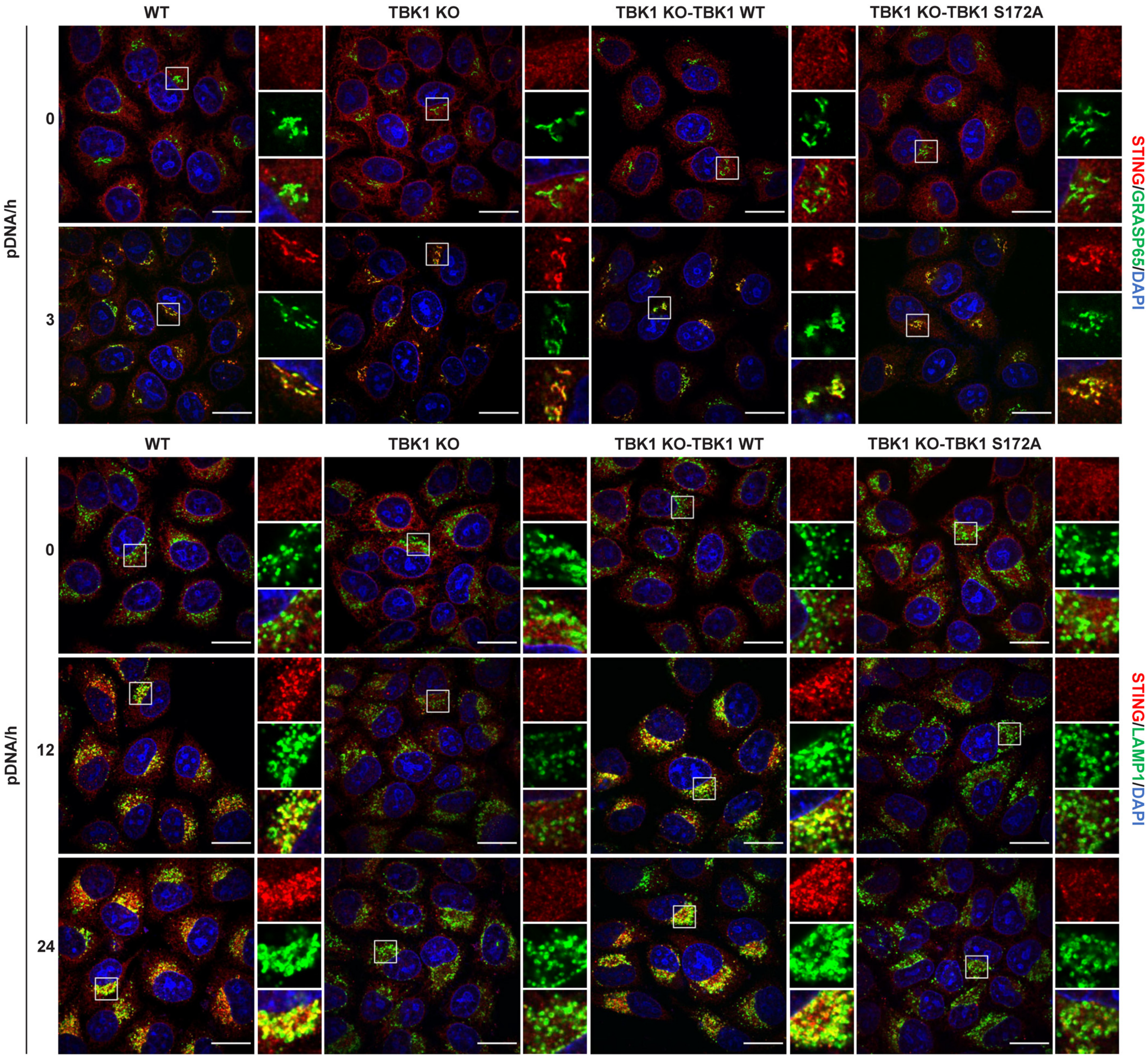

Figure EV7. TBK1 controls STING post-Golgi trafficking via its kinase activity.

TBK1-knockout HeLa cells, *TBK1*-knockout HeLa cells stably expressing WT TBK1 (TBK1 KO-TBK1 WT) and kinase-dead mutants (TBK1 KO-TBK1 S172A) and WT HeLa cells were stimulated with pDNA transfection (1 µg/mL) for 3, 12 and 24 h, followed by confocal imaging to observe STING colocalization with GRASP65 and LAMP1, respectively; scale bars, 20 µm. Source data are available online for this figure.

 © The Author(s)

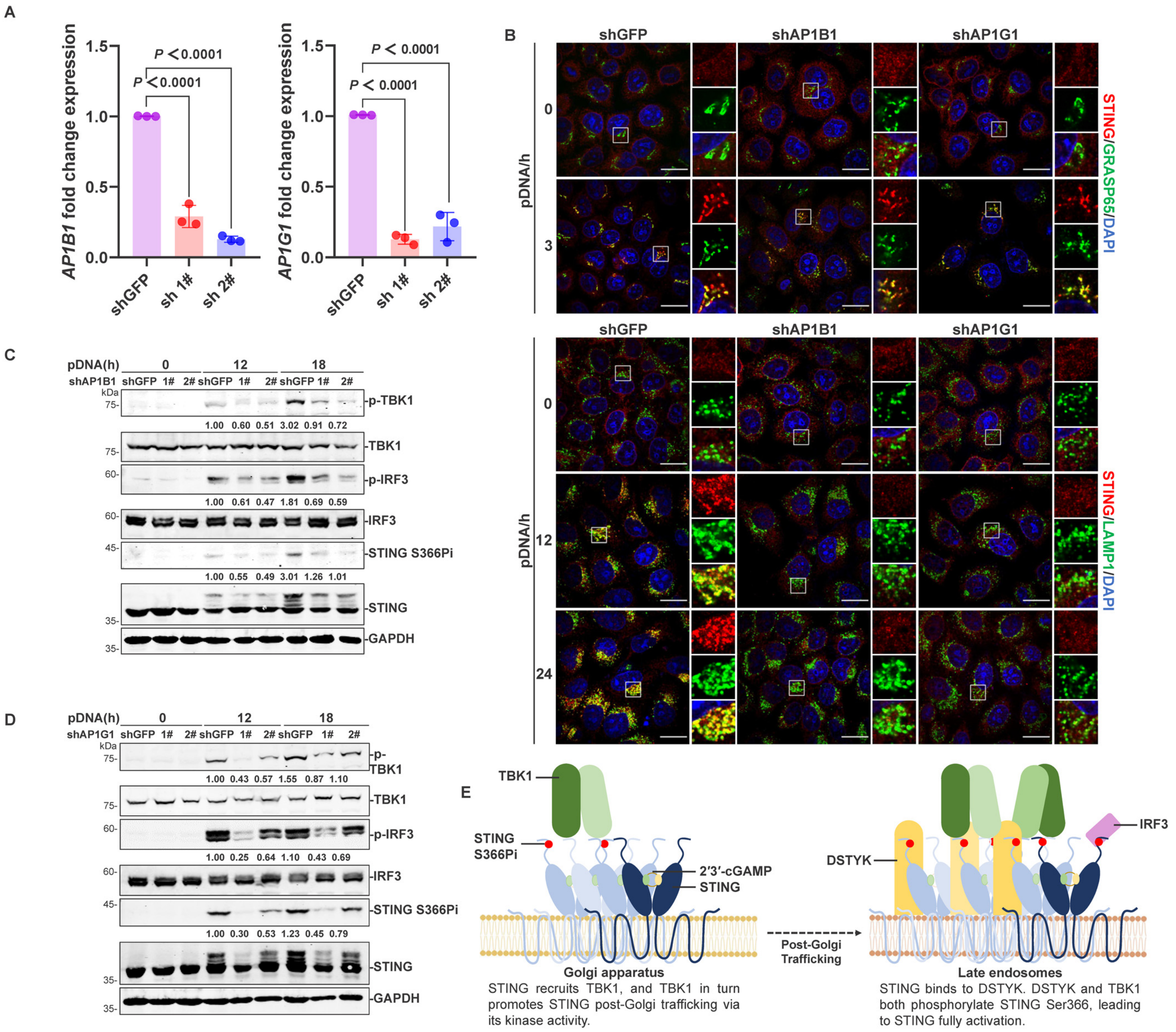

Figure EV8. Blocking STING post-Golgi trafficking inhibits STING signaling activation, and the schematic diagram.

(**A**) Detection of *AP1B1* and *AP1G1* knockdown by qPCR ($n = 3$ biological replicates). (**B**) AP-1 deficiency inhibit STING colocalization with LAMP1. *AP1*-knockdown HeLa cells and control cells (shGFP) were stimulated with pDNA (1 μg/mL) for 3, 12, and 24 h to observe STING and GRASP65 colocalization as well as STING and LAMP1 colocalization, respectively, by confocal imaging; scale bars, 20 μm. (**C**, **D**) AP-1 deficiency inhibit pDNA transfection-induced phosphorylation of TBK1, IRF3, and STING at Ser366. *AP1*-knockdown HeLa cells and control cells (shGFP) were stimulated with pDNA (1 μg/mL) for the indicated lengths of time to observe phosphorylation of TBK1, IRF3, and STING at Ser366 via western blotting. Quantification of the indicated band intensities was performed in ImageJ, and quantification results are labeled below the indicated bands ($n = 3$ technical replicates). (**E**) The schematic diagram to summarize the working model. Activated STING arrives the Golgi and recruits TBK1, and TBK1 recruitment promotes STING post-Golgi trafficking to late endosomes via its kinase activity. TBK1 and late endosomal-localized DSTYK can both directly phosphorylate late endosomal-localized STING at Ser366, thus further promoting maximized activation of STING signaling. Data are representative of at least three independent experiments. Data in (**A**) are shown as the mean ± SD and *P* values were analyzed by one-way ANOVA and the exact *P* values are shown in the figures.
